# Crosstalk between astrocytes and microglia results in increased degradation of α-synuclein and amyloid-β aggregates

**DOI:** 10.1186/s12974-021-02158-3

**Published:** 2021-06-03

**Authors:** Jinar Rostami, Tobias Mothes, Mahshad Kolahdouzan, Olle Eriksson, Mohsen Moslem, Joakim Bergström, Martin Ingelsson, Paul O’Callaghan, Luke M. Healy, Anna Falk, Anna Erlandsson

**Affiliations:** 1 Molecular Geriatrics, Rudbeck Laboratory, Department of Public Health & Caring Sciences/, Uppsala University, Uppsala, Sweden; 2 Department of Neurology and Neurosurgery, Montreal Neurological Institute, McGill University, Montreal, Canada; 3 Department of Medical Cell Biology, Uppsala University, Uppsala, Sweden; 4 Department of Neuroscience, Karolinska Institutet, Stockholm, Sweden

**Keywords:** Alzheimer’s disease, Parkinson’s disease, α-Synuclein, Crosstalk, Amyloid-β, Astrocyte, Microglia, Co-culture, Degradation, Tunneling nanotube

## Abstract

**Background:**

Alzheimer’s disease (AD) and Parkinson’s disease (PD) are characterized by brain accumulation of aggregated amyloid-beta (Aβ) and alpha-synuclein (αSYN), respectively. In order to develop effective therapies, it is crucial to understand how the Aβ/αSYN aggregates can be cleared. Compelling data indicate that neuroinflammatory cells, including astrocytes and microglia, play a central role in the pathogenesis of AD and PD. However, how the interplay between the two cell types affects their clearing capacity and consequently the disease progression remains unclear.

**Methods:**

The aim of the present study was to investigate in which way glial crosstalk influences αSYN and Aβ pathology, focusing on accumulation and degradation. For this purpose, human-induced pluripotent cell (hiPSC)-derived astrocytes and microglia were exposed to sonicated fibrils of αSYN or Aβ and analyzed over time. The capacity of the two cell types to clear extracellular and intracellular protein aggregates when either cultured separately or in co-culture was studied using immunocytochemistry and ELISA. Moreover, the capacity of cells to interact with and process protein aggregates was tracked using time-lapse microscopy and a customized “close-culture” chamber, in which the apical surfaces of astrocyte and microglia monocultures were separated by a <1 mm space.

**Results:**

Our data show that intracellular deposits of αSYN and Aβ are significantly reduced in co-cultures of astrocytes and microglia, compared to monocultures of either cell type. Analysis of conditioned medium and imaging data from the “close-culture” chamber experiments indicate that astrocytes secrete a high proportion of their internalized protein aggregates, while microglia do not. Moreover, co-cultured astrocytes and microglia are in constant contact with each other via tunneling nanotubes and other membrane structures. Notably, our live cell imaging data demonstrate that microglia, when attached to the cell membrane of an astrocyte, can attract and clear intracellular protein deposits from the astrocyte.

**Conclusions:**

Taken together, our data demonstrate the importance of astrocyte and microglia interactions in Aβ/αSYN clearance, highlighting the relevance of glial cellular crosstalk in the progression of AD- and PD-related brain pathology.

**Supplementary Information:**

The online version contains supplementary material available at 10.1186/s12974-021-02158-3.

## Background

The impact of glial cells on pathological processes in various neurodegenerative diseases has recently received much attention. Glial crosstalk is crucial for maintaining brain homeostasis and is believed to play a key role in chronic neuroinflammation. The conversation between microglia and astrocytes starts already during early development and remains throughout life [1]. However, exactly in which way the crosstalk between the two cell types affects the progression of neurodegenerative diseases remains unclear.

Alzheimer’s disease (AD) and Parkinson’s disease (PD) are the two most common neurodegenerative disorders, characterized by cognitive and functional impairments, which develop over many years. The major pathological hallmarks of the diseases are accumulation of toxic protein aggregates. In PD, intracellular inclusions of alpha-synuclein (αSYN) are found in neurons and glia, while both extracellular and intracellular deposits of amyloid-beta (Aβ) are present in the AD brain [2, 3]. Other common features of AD and PD are neuronal cell death and chronic neuroinflammation, involving both innate and adaptive immune responses [4].

In the healthy adult brain, astrocytes are rather stationary cells, forming a well-coordinated network in the parenchyma [5]. Microglia, on the other hand, are motile and constantly sensing the environment around them [6, 7]. In the AD and PD brain, both astrocytes and microglia acquire reactive, inflammatory phenotypes and can effectively phagocytose aggregated proteins and cell debris, as well as secrete various cytokines and inflammatory mediators [8–13]. Up until now, most research has focused on the role of each cell type by itself. However, recent data indicate that microglia and astrocytes coordinate their functions in many ways, both in the healthy and diseased brain [14–21].

Chronic glial activation may contribute to AD/PD progression in several ways, including loss of homeostatic function and gain of toxic functions, causing neuronal loss [22]. In addition, reactive glial cells may be responsible for cell-to-cell spreading of the pathology. We have previously reported that cultured astrocytes accumulate αSYN and Aβ aggregates but not the monomeric form of the proteins [11, 12]. The αSYN and Aβ accumulation result in severe cellular stress, indicated by lysosomal and mitochondrial deficiencies [11, 12, 23]. Subsequently, stressed astrocytes respond by sending out tunneling nanotubes (TNTs), enabling intercellular transfer of pathogenic protein aggregates, as well as MHCII complexes to nearby cells [11, 24]. The aim of the present study was to investigate how microglia and astrocytes coordinate their responses in regard to αSYN and Aβ pathology, including accumulation and degradation of protein aggregates, and cell-to-cell contacts.

## Methods

### Production of alpha-synuclein fibrils

Alpha-synuclein preformed fibrils (αSYN-F) were generated using endotoxin-free monomeric αSYN (AnaSpec, A5555-1000) as described previously [24]. Shortly, the monomers were dissolved in PBS at a concentration of 5mg/ml and left on a shaker at 1000 rpm for 7d. Thereafter, αSYN-F were diluted to a working concentration of 2mg/mL in PBS and stored at −80 °C until use. The αSYN-F were Cy3-labelled using Cy3^AM^ antibody labelling kit (GE Healthcare, PA33000) as described previously [24]. Prior to each experiment, the αSYN-F were diluted 1:2 in PBS and sonicated twice in 20% amplitude, 1s off and 1s on, for 30s using a Sonics Vibra Cell sonicator.

### Production of amyloid-beta fibrils

Amyloid-beta preformed fibrils (Aβ-F) were generated using human Cy3-labelled Aβ-42 monomers (AnaSpec, 60480-01). The Aβ monomers were dissolved in a 10 mM NaOH/PBS solution to a concentration of 2 mg/ml. The Aβ samples were left to aggregate on a shaker at 1500 rpm, 37 °C for 4d. Then, the Aβ-F were diluted using peptide PBS to the final concentration of 0.5 mg/ml and sonicated in 20% amplitude, 1s off and 1s on, for 30s using a Sonics Vibra Cell sonicator.

### Culture of human iPSC-derived astrocytes

Human astrocytes were generated from neuroepithelial-like stem (NES) cells, produced from human-induced pluripotent stem cells (iPSCs, Cntrl9 cell line) [25, 26]. The NES cells were differentiated in Advanced DMEM/F12 (Thermo Fisher 12634-010) supplemented with 1% penicillin–streptomycin (Thermo Fisher 15140-122), 1% B27 supplement (Thermo Fisher, 11530536), 1% non-essential amino acids (Thermo Fisher, 11140050), and 1% l-glutamine (Thermo Fisher 25030-024). The following factors were added fresh to the medium just before use: basic fibroblast growth factor (bFGF) 10 ng/ml (Themo Fisher, 13256029), heregulin 10 ng/ml (Sigma Aldrich, SRP3055), activin A 10 ng/ml (Peprotech, 120-14E), and insulin-like growth factor 1 (IGF-1) 200 ng/ml (Sigma Aldrich, SRP3069). Additionally, 20 ng/ml ciliary neurotropic factor (CNTF; Thermo Fisher, PHC7015) was added to the medium the last 2 weeks of differentiation. Cells were seeded for experiment, at a concentration of 5000 cells/cm^2^, directly after the differentiation protocol was completed.

### Culturing of human iPSC-derived microglia

To enable co-cultures of human iPSC-derived astrocytes and microglia without strain-induced immunological reactions, microglia were generated from the same human iPSC line that was used for deriving the NES cells. Human iPSCs (Cntrl9 cell line) were cultured in mTeSR Plus medium (Stemcell Technologies, 05825) on matrigel (VWR, 356234)-coated 6-well plates and passaged when an 80% cell density was reached. The iPSCs were passaged using ReLeSR (Stemcell Technologies, 05872), according to the manufacturer’s instructions. Generation of hematopoietic progenitor cells (HPCs) and further differentiation to microglia were performed according to the previously published protocol [27], with a few modifications. In short, human iPSCs were seeded at a very low density, and the following day, the mTeSR medium was replaced with medium A (STEMdiff Hematopoietic basal medium supplemented with 1:200 supplement A) (Stemcell Technologies, 05310). After 2d, half of the medium was replaced with fresh medium A. On d3, medium A was completely removed, and medium B (STEMdiff Hematopoietic basal medium supplemented with 1:200 supplement B) was added to the wells. Every other day, 1 ml fresh medium B was added to the cells. On d14, hematopoietic progenitor cells (HPCs) were collected and analyzed with flow cytometry (Fortessa) for the following HPC markers: APC anti-human CD43 (BD Biosciences, 343206) and PE anti-human CD41 (Nordic Biosite, 303706). In addition,the HPCs were stained for Zombie Violet viability dye (Nordic Biosite, 423114) for viability check prior to seeding on matrigel-coated plates. Microglia maturation medium consisted of DMEM F12 (Thermo Fisher, 11039021) supplemented with 2X insulin-transferrin-selenite (Thermo Fisher, 41400045), 2X SM1 (Stemcell Technologies, 5711), 0.5X N2 (Thermo Fisher, 17502048), 1X glutamax (Thermo Fisher, 35050061), 1X non-essential amino acids (Thermo Fisher, 11140050), 400 μM monothioglycerol (Sigma Aldrich, M1753), and 5 μg/mL insulin (Sigma Aldrich, I2643). In addition, the following factors were added to the medium just before use: IL34 (Peprotech, 200-34, 100 ng/mL), MCSF (Peprotech, 300-25, 25ng/mL), IDE1 (Peprotech, 1164899, 10μg/ml). On d24 of microglial differentiation, two additional factors, CX3CL1 (Peprotech, 300-31, 100 ng/mL) and CD200 (Bonopus, C311, 100 ng/mL), were included in the microglia maturation medium. Microglia were plated at the same seeding density as astrocytes (5000 cells/cm^2^) and used for experiments within 14d after completed differentiation.

### Co-culture of human iPSC-derived microglia and astrocytes

Fully differentiated microglia cells were cultured on matrigel-coated 6-well plates (25,000 cells/well) and left there to adapt for 3d before astrocytes (25,000 cells/well) were added to the culture. Microglia maturation medium supplemented with all the factors both cell types require was used for the co-culture system. Three days after seeding of the astrocytes, co-cultures were exposed to the different treatments.

### Exposure to αSYN-F and Aβ-F

Astrocytes, microglia, and co-cultures were exposed to 0.5 μM αSYN-F or 0.2 μM Aβ-F for 24h, 4d, or 7d. The concentrations were selected based on our previous studies [11, 12, 23, 28, 29]. The lower concentration of Aβ was used since aggregated Aβ is more toxic to astrocytes than aggregated αSYN. Cell culture media samples from all time points were collected and stored at −80 °C. Additional time-points that were included for the two treatments are described below.

#### αSYN-F

Astrocytes, microglia, and co-cultures were studied at 24h+3d and 24h+6d. After 24h of exposure, cells were washed twice with medium and cultured in αSYN-free medium for additional 3d or 6d.

#### Aβ-F

Astrocytes were studied at 4d+3d. Four days after Aβ-F exposure, astrocytes were washed twice with medium and cultured for additional 3d in Aβ-free medium. Co-cultures were studied at 24h+3d, 24h+6d, and 4d+3d following Aβ-F exposure.

### Conditioned media experiments

For conditioned media experiments, the astrocytes and the microglia cells were treated with αSYN-F or Aβ-F for 24h. Thereafter, the cells were washed and kept in culture for additional 3d (24h+3d). The conditioned medium from the astrocytes was diluted 1:1 in microglia medium before addition to untreated microglia cultures for 24h.Thereafter, the medium was collected, and cells were fixed. Similarly, conditioned medium from the 24h+3d time point microglia cells was collected and diluted 1:1 in astrocyte medium and added to untreated astrocyte cultures for 24h before medium collection and fixation.

### “Close-culture” chamber system

The close-culture chamber system was designed to hold two coverslips in close proximity to each other. The device was drawn in Fusion360 (Autodesk Inc, CA, USA) and milled from 316L steel using a computer numerical controlled mill. The device consisted of two interlocking steel rings. A 24-mm diameter coverslip was placed at the base of the bottom ring on which one monoculture was established and submerged in medium in a cell culture dish. The second mono-culture was established on an 18-mm diameter coverslip, which was placed upside-down on a flange on the upper side of the bottom ring. The flange was 1-mm thick ensuring the coverslips were separated by this distance. The top ring was then positioned to complete the close-culture sandwich and ensure the upper coverslip was weighted in place for the duration of the experiment. Three access ports in the inner culture chamber, formed by the interconnected steel rings, facilitated medium exchange.

The bottom coverslip with astrocytes or microglia was exposed to 0.5 μM αSYN-F for 24h and thoroughly washed prior to the assembly of the chamber. A mixture of microglia and astrocyte medium (1:1) was added, and the untreated astrocytes/microglia coverslip was placed in the chamber upside down. The untreated cells (top coverslip) were co-cultured with αSYN-F exposed cells (bottom coverslip) for 3d and then dissembled and fixed for analyses.

### Immunocytochemistry

Cells were fixed in 4% paraformaldehyde (PFA) (Sigma Aldrich) in PBS and washed twice with PBS. Blocking and permeabilization were performed with 5% normal goat serum (NGS) (Bionordika, S-1000) and 0.1% triton in PBS for 30 min in room temperature (RT). Primary antibodies were diluted in 0.5% NGS and 0.1% triton in PBS and added to the cells overnight (ON) at +4 °C. Thereafter, cells were washed 3 times with PBS prior to incubation with secondary antibodies or dyes for 1h at RT. After additional washes, the cells were mounted, using Ever Brite Hardset Mounting medium with or without DAPI (BioNordika). Images were captured using a fluorescence microscope Observer and Z1 Zeiss. The primary antibodies used were chicken anti-vimentin antibody (Sigma Aldrich, AB5733), mouse anti-S100B antibody (Sigma Aldrich, S2532), Rabbit anti-ALDH1L1 (Novus biologicals, NBP2-24143), Chicken anti-GFAP (Abcam, ab4674), Rabbit anti-Iba1 antibody (Abcam, ab178846), Rabbit anti-P2Y12 antibody (Thermo Fisher, 702516), Mouse anti-CD68 (Abcam, ab955), and mouse anti-LAMP1 antibody (Abcam, ab25630). To stain the plasma membrane, cells were incubated with wheat germ agglutinin (WGA) Alexa Fluor® 350 Conjugate (Life technologies, 1:100) together with the primary antibodies ON. The secondary antibodies used were Alexa Flour 488 goat anti rabbit/mouse (Thermo Fisher, 1:200) and Alexa Flour 647 goat anti rabbit/mouse (Thermo Fisher, 1:200).

### Time-lapse microscopy

Cells were recorded using a time-lapse microscopy (Nikon Biostation IM Cell Recorder). Images were taken every 10 min with ×20 and ×40 objectives. The duration of the experiment was 24h+3days to 24h+6days.

### ELISA

EIA/RIA half area 96-well plates were used for all the ELISAs. Antibodies diluted in PBS were used for coating the plates ON at +4 °C. Then, the plates were blocked with 1% BSA in PBS at RT for 3h on shake at 900 rpm. ELISA incubation buffer (0.2% tween, 0.1% BSA, and 0.15% Kathon) was used to prepare standards, samples and antibody solutions, which were then added to the plates and incubated ON at 4 °C on shake (900 rpm). The next day, the plates were incubated with detection antibody for 2h at RT on shake and washed. The signal was developed using K-blue aqueous for 7 min, and 1M H_2_SO4 was added to the plates to stop the reaction. Plates were read at 450nm using an Infinite M200 Pro.

#### Total αSYN

For the αSYN ELISA, MJFR1 (0.25μg/ml in PBS) (Abcam, 138501) was used as the coating antibody, and synthetic monomeric αSYN (Proteos) was used as a standard for total αSYN detection. Prior to analysis, the 0-time point medium samples (containing 0.5μM αSYN-F) were diluted 1:50, the 24h time-point samples were diluted 1:10, and samples from the later time-points were left undiluted. Biotinylated anti-αSYN antibody clone 42 (BD Biosciences 610787, 0.35μg/ml) was used as detection antibody, followed by anti-mouse F(ab)2-HRP (1:2000, Jackson) incubation for 1h in RT on shake.

#### Total Aβ

Anti-Aβ_42_ antibody (Invitrogen 2μg/ml) was used as the coating antibody, and synthetic Aβ_42_ was used as a standard. The medium samples, as well as the standards, were denatured in 0.5% sodium dodecyl sulfate (SDS) at 90 °C for 5min, followed by a 1:10 dilution (in order to lower the SDS concentration to 0.05% SDS). Biotinylated mAb4G8 (Nordic Biosite, 80070) was used as detection antibody, followed by incubation with SA-HRP (1:2000 Mabtech) for 1h at RT.

### Cytokine assay

Medium samples from astrocyte, microglia, and co-cultures were treated with αSYN-F and Aβ-F and collected and analyzed at 7d. Samples from microglia and astrocytes treated with 100ng/ml LPS for 24h was included as positive controls. The cytokine array (R&D systems, ARY005B) was performed according to the manufacturer’s protocol. Shortly, the membranes were blocked with array buffer 4 at the same time as the samples were incubated with the detection antibody cocktail for 1 h on shake at RT. Next, the antibody-sample mixture was added to the membranes, which were incubated ON at 4 °C. The following day, the membranes were washed with the washing buffer 3 × 10 min and incubated with HRP (1:1000) for 30 min before additional washes. The membranes were then incubated with a mixture of reagent 1 and 2 (1:1) for 10 min and developed in the chemiluminescent machine for 30s.

### Image analysis

#### Immunocytochemistry

The Z-layers of the αSYN respective Aβ channels were fused using ImageJ, and the mean integrated density (IntDen) (area*mean intensity) was measured. In both the separate cultures and in the co-cultures, microglia and astrocytes were outlined using the cellular markers Iba1 and vimentin, limiting the analysis to internalized αSYN/Aβ only. The IntDen αSYN/Aβ signal was measured within the outlined cells. Since the astrocytes and microglia proliferated during the time course of the experiment (Supplementary Figure 1 and 2), we normalized the IntDen αSYN/Aβ signal to the area and not to the cell number. In proliferating cultures, normalization to the cell number is misleading as the deposits per cell will then be reduced without any degradation taking place.

#### Cytokine assay

An oval selection was generated as the region of interest. Mean intensity of each dot corresponding to a cytokine was measured using the oval selection. Medium samples from three independent experiments were analyzed.

### Statistical analysis

The level of significance for all the graphs was as follows: * = *P* < 0.05, ** = *P* < 0.01, *** = *P* < 0.001, and **** = *P* < 0.0001.

#### Image analysis

Sixty Z-stack images per time point and treatment for each culture system were captured from three independent experiments. The 4d, 7d, 24h+3d, and 24h+6d time points were normalized to the 24h time-point, and Kruskal-Wallis statistical analysis with Dunn’s correction was performed as the groups failed to pass normality.

#### αSYN and Aβ measurements in ELISA

Medium samples from four different experiments were used for all ELISAs. The 0-time point was set to 100%, and all the other time points were normalized to the 0-time point, and one-way ANOVA with Tukey correction was performed.

## Results

### Astrocytes, but not microglia, accumulate αSYN-F over time

**Fig. 1:**
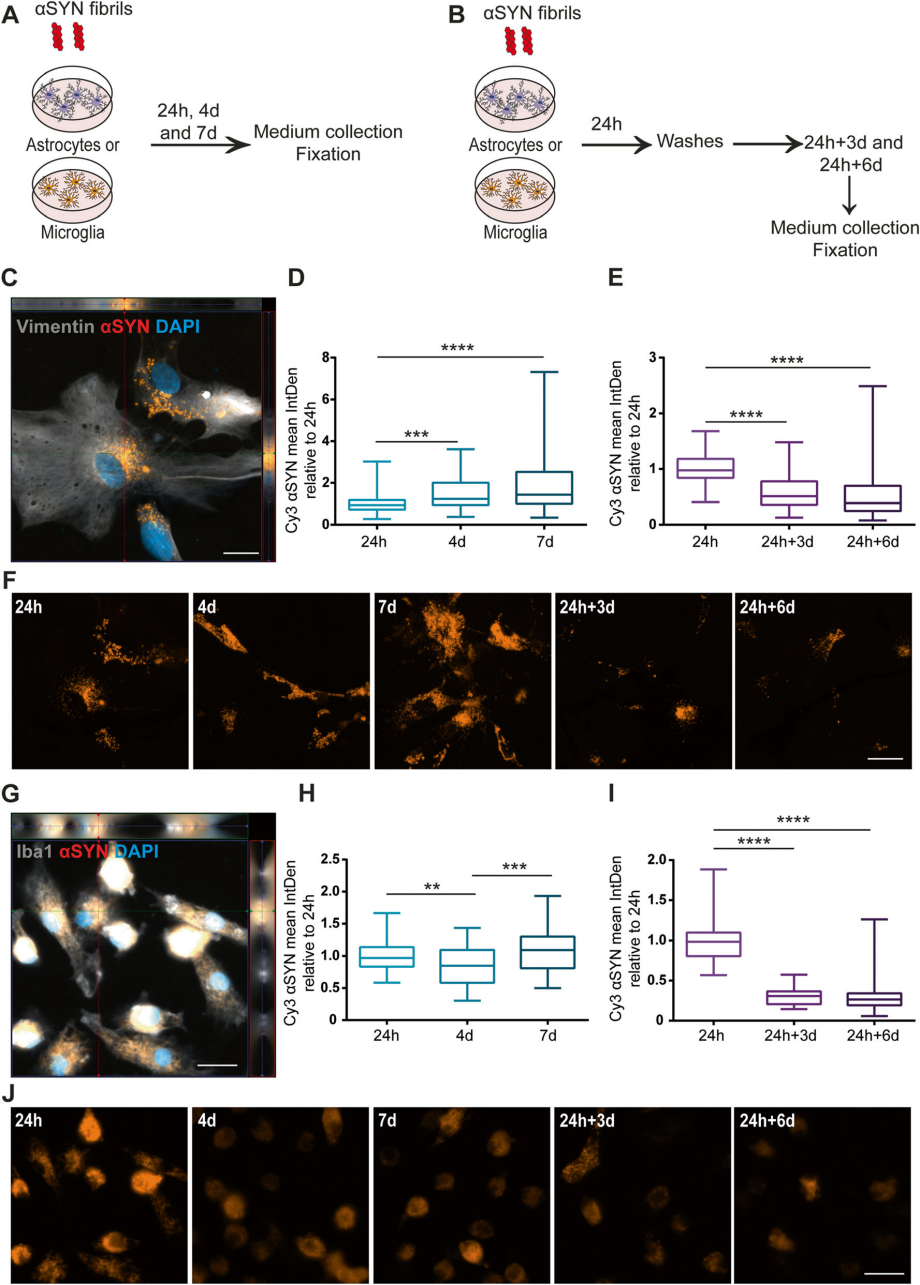
In contrast to microglia, astrocytes accumulate αSYN over time. Schematic figure of the study design illustrating that the cells were treated with αSYN-F, either continuously (**a**) or with a 24h pulse (**b**). Astrocytes ingested and accumulated αSYN-F already at 24h (**c**). However, the accumulation was continuous over the course of 7d, resulting in an increased intracellular signal of αSYN (**d**). However, when the cells were washed at 24h and cultured without αSYN-F for 3d and 6d, a significant reduction was observed over time (**e**). Representative images of the αSYN deposits in astrocytes at the different time points are shown in **f**. Microglia showed extensive amounts of intracellular αSYN-F at 24h (**g**). However, microglia did not show the same level of accumulation over time as compared to the astrocytes but had unchanged levels of intracellular αSYN from 24h to 7d (**h**). Only low levels of αSYN were present in microglia at 24h+3d and 24h+6d, indicating more effective degradation in microglia than in astrocytes (**i**). Representative images of the αSYN deposits in microglia at the different time points are shown in (**j**). Scale bars = 20μm

Human astrocytes, expressing the astrocytic markers vimentin, S100β, ALDH1L1 and GFAP (Supplementary Figure 3 A), and human microglia, expressing the microglial markers Iba1 and P2Y12 (Supplementary Figure 3 B) were derived from the same hiPSC line [27]. To compare the engulfment and accumulation of αSYN-F by the astrocytes and microglia, the two cell types were cultured separately and exposed to αSYN-F for 24h, 4d, and 7d (Fig. 1a). To be able to draw conclusions about the degradation capacity of the cells, parallel cultures were exposed to an αSYN-F pulse and then cultured for additional time following removal of αSYN-F from the medium (Fig. 1b). Already at 24h, Cy3-labelled intracellular αSYN was detected in astrocytes, indicating uptake of αSYN-F (Fig. 1c). Image analysis revealed that when the astrocytes were treated with αSYN-F over the course of 7d, the intracellular αSYN signal gradually increased (Fig. 1d). However, upon removal of extracellular αSYN-F, the intracellular αSYN signal decreased, indicating that the astrocytes were capable of degrading at least a proportion of the ingested αSYN-F (Fig. 1e). Representative images of the αSYN deposits in astrocytes at the different time points are shown in Fig. 1f. Similar to astrocytes, microglia showed a robust uptake of αSYN-F at 24h (Fig. 1g). In contrast to the astrocytes, no increase was observed in the microglia cells over time. Instead, there was no significant change of intracellular αSYN in the culture between the 24h and 7d time points (Fig. 1h). Upon extracellular αSYN-F-removal, the intracellular αSYN signal was dramatically reduced, indicating that microglia are more effective than astrocytes when it comes to degrading the ingested αSYN-F (Fig. 1i). Notably, intracellular αSYN was still observed in both monocultures at 24h+6d, indicating that some deposits are more difficult for the cells to clear (Fig. 1e and i). Representative images of the αSYN deposits in microglia at the different time points are shown in Fig. 1j. Since microglia continued to proliferate over the 7d, the αSYN signal per cell was reduced over time, although the total αSYN content in the culture was unchanged (Fig. 1h–j and Supplementary Figure 2).

### Co-culturing of microglia and astrocytes results in reduced αSYN accumulation

**Fig. 2:**
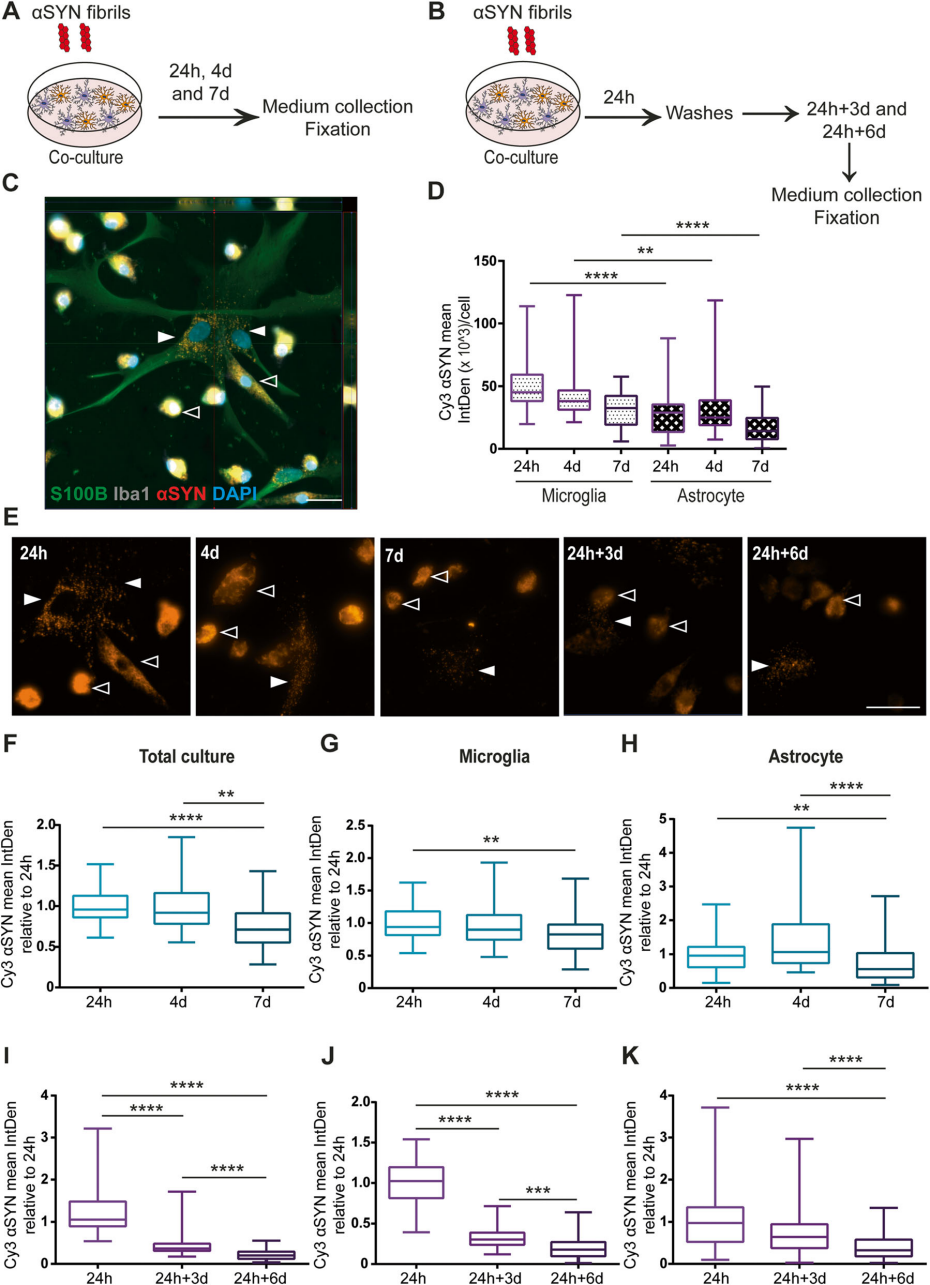
αSYN accumulation is reduced when microglia and astrocytes are co-cultured. Schematic figure of the study design illustrating that the cells were either treated with αSYN-F constantly (**a**) or with a 24h pulse (**b**). Both astrocytes (Iba1- S100B+, filled arrow heads) and microglia (Iba1+, open arrow heads) ingested and accumulated αSYN in the co-culture set-up (**c**). Image analysis showed that the microglia contained more αSYN signal per cell at 24h and 4 d, compared to the astrocytes (**d**). Representative images of the αSYN-F deposits at the different time points in the co-culture are shown in **e**; astrocytes and microglia are indicated with filled respective open arrow heads. Analysis of the total intracellular αSYN in the co-culture revealed that there was an overall reduction of αSYN at d7 in relation to the 24h time point (**f**). Separate image analysis of the αSYN content in the two cell types of the co-culture confirmed that the decrease in αSYN signal was significantly lower in both microglia (**g**) and astrocytes (**h**) at d7. For the astrocytes, the reduction in αSYN was opposite to the pattern in the pure astrocytic culture, suggesting a lower accumulation of αSYN within astrocytes in the co-culture. When the co-cultures were washed at 24h and cultured without αSYN-F for 3d and 6d, a significant reduction was observed over time in the total co-culture (**i**), which was due to a decrease in both co-cultured microglia (**j**) and co-cultured astrocytes (**k**). Scale bars = 20μm

Next, we investigated αSYN-F uptake and accumulation in a microglia and astrocyte co-culture system. Similar to the monocultures, the co-cultures were either exposed to αSYN-F continuously for 24h, 4d, and 7d (Fig. 2a) or received an αSYN-F pulse (Fig. 2b). Using specific cell markers, we were able to analyze the intracellular αSYN signal in the microglia and astrocytes, respectively (Fig. 2c). By normalizing αSYN IntDen to the total number of respective cell type, we revealed that microglia cells contained significantly more αSYN per cell at 24h and 4d, compared to the astrocytes (Fig. 2d and e and Supplementary Figure 4).

To find out if the co-existence of the two cell types affected the overall αSYN accumulation, the total αSYN signal in culture was analyzed. Interestingly, the total αSYN signal was significantly reduced at 7d (Fig. 2f). This was in contradiction to the results in the monocultures, where αSYN signal was increased at 7d in the astrocytes and unchanged in the microglia cultures (Fig. 1d and h). Hence, the presence of both microglia and astrocytes reduces the overall accumulation of αSYN. To identify which cell type that was mainly responsible for the reduction, microglial and astrocytic αSYN content was analyzed. Intracellular αSYN signal appeared to be mostly reduced in astrocytes at 7d (Fig. 2g and h). In line with the results from the monocultures, there was a clear reduction of intracellular deposits in the co-culture set-up, following removal of αSYN (24h+3d and 24h+6d, Fig. 2i–k), although there were some remaining deposits that were not cleared.

### Intracellular Aβ levels remain stable in astrocytes, but decrease over time in microglia

**Fig. 3:**
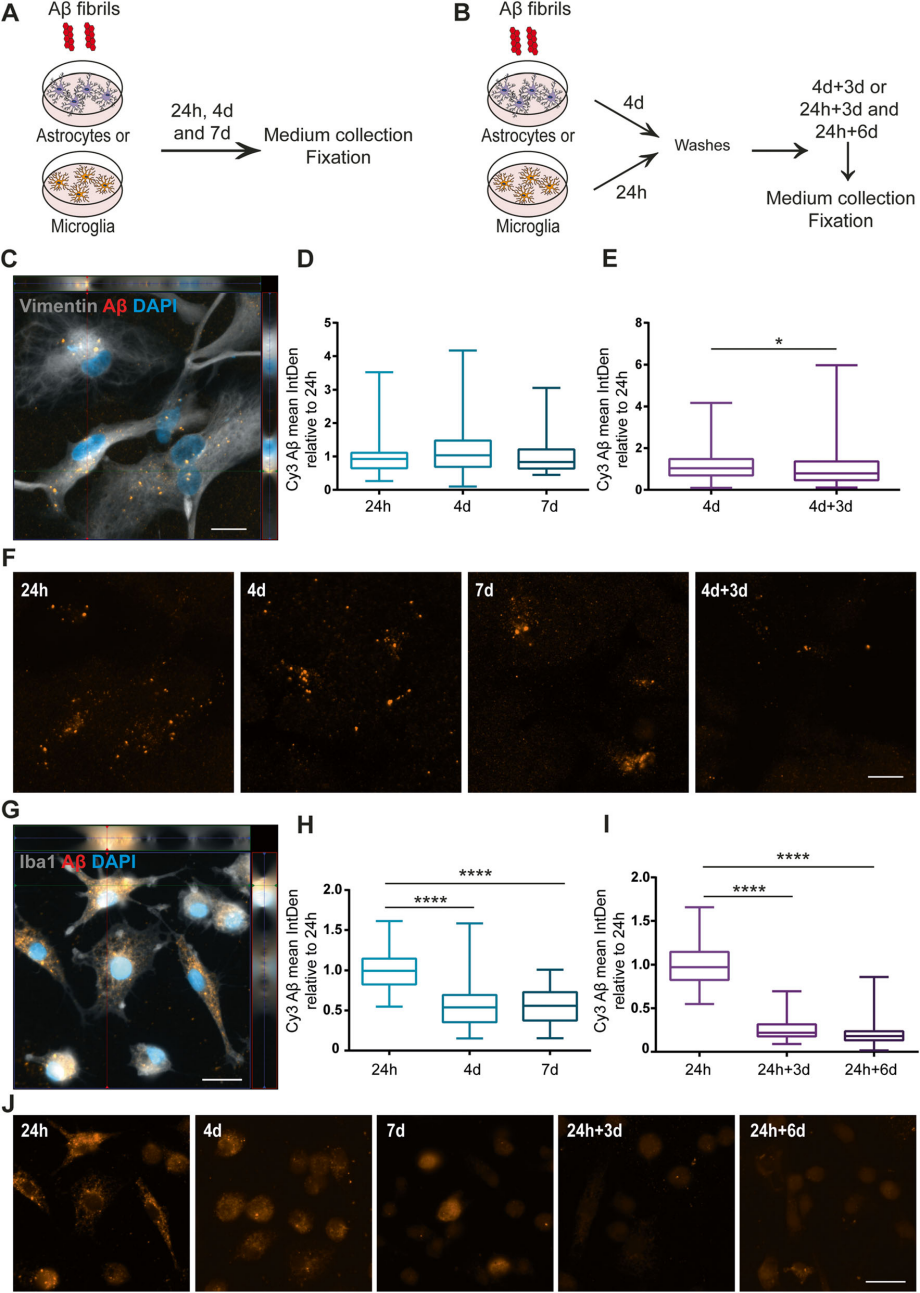
Intracellular Aβ levels are reduced in microglia over time but remain stable in astrocytes. Schematic figure of the study design illustrating that the cells were either constantly treated with Aβ-F for 24h, 4d, and 7d (**a**) or treated with an Aβ-F pulse (**b**), lasting for 24h (microglia) or 4d (astrocytes), followed by culture in Aβ-F-free medium. Astrocytes had ingested and accumulated Aβ-F already at 24h (**c**). The intracellular Aβ accumulation was significantly increased after 4d compared to 24h (**d**). At 4d+3d, the astrocytic Aβ signal was significantly reduced, compared to the 4d time point (**e**). Representative images of the Aβ deposits in astrocytes at the different time points are shown in **f**. Large amounts of intracellular Aβ could be detected in microglia at 24h (**g**). Contrary to the astrocytes, microglia showed a reduction in intracellular Aβ signal at 4 days and 7d compared to 24h (**h**). Furthermore, the intracellular Aβ aggregates in microglia were significantly lowered at 24h+3d and 24+6d, compared to 24h (**i**). Representative images of the Aβ deposits in microglia at the different time points are shown in **j**. Scale bars = 20μm

Similar to the αSYN-F experiments, monocultures of astrocytes and microglia were treated with Aβ-F to study intracellular accumulation and degradation (Fig. 3a, b). The intracellular levels of Aβ-F at 24h in astrocytes appeared lower than the levels of αSYN-F (Fig. 3c). However, direct comparison of the Cy3-intensity could not be done, since the efficiency of Cy3-labelling of Aβ and αSYN may differ. Furthermore, no significant difference in intracellular Aβ inclusions could be detected between 24h and 7d (Fig. 3d). At 4d+3d, the astrocytic Aβ signal was slightly reduced, compared to the 4d time point, indicating that astrocytes were capable of degrading the ingested Aβ to some degree (Fig. 3e). Representative images of the Aβ deposits in astrocytes at the different time points are shown in Fig. 3f. The microglia cells showed an extensive uptake of Aβ-F aggregates at 24h (Fig. 3g). In contrast to αSYN, the intracellular Aβ signal was significantly decreased at 4d. On the other hand, no change in intracellular Aβ was observed from 4 to 7d (Fig. 3h). Although the intracellular Aβ aggregates were significantly lowered at 24h+3d and 24h+6d, there were still some remaining Aβ deposits at these time points, indicating that the microglia were not capable of complete degradation during the 6d time span (Fig. 3i). Representative images of the Aβ deposits in microglia at the different time points are shown in Fig. 3j.

### Astrocytes contain less Aβ deposits when co-cultured with microglia

**Fig. 4:**
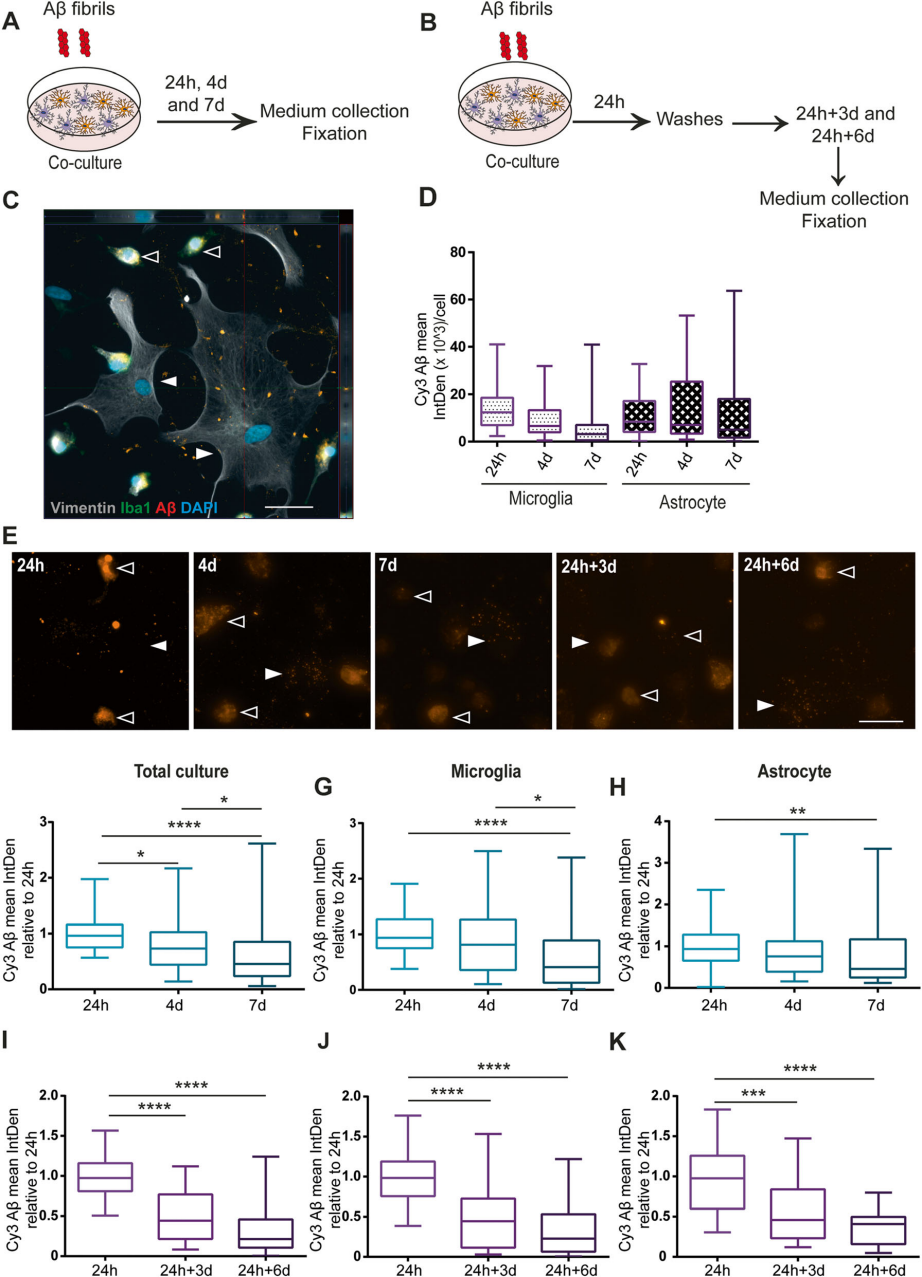
Intracellular Aβ is reduced when astrocytes and microglia are cultured together. Schematic figure of the study design illustrating that the cells were either treated with Aβ-F constantly (**a**) or with a 24h Aβ-F pulse (**b**). In the co-culture, both astrocytes (Iba1- S100B+, filled arrow heads) and microglia (Iba1+, open arrow heads) ingested and accumulated Aβ-F (**c**). Image analysis and normalization to the number of cells confirmed that astrocytes and microglia contained comparable levels of Aβ at 24h, 4d and 7d (**d**). Representative images of the Aβ deposits at the different time points in the co-culture are shown in (**e**); astrocytes and microglia are indicated with filled respective open arrow heads. Analysis of the total intracellular Aβ in the co-culture revealed that intracellular Aβ was lower in the culture at 7d, compared to 24h (**f**). Separate analysis of the two cell types in the co-culture demonstrated that the astrocytes, rather than the microglia, were responsible for this reduction (**g**, **h**). When the co-cultures were washed at 24h and cultured without Aβ-F for 3d and 6d, a significant reduction was observed over time (**i**), which was due to a decrease in both microglia (**j**) and astrocytes (**k**). Scale bars = 20μm

To examine whether culturing microglia and astrocytes together also decreased Aβ accumulation, co-cultures were exposed to Aβ-F continuously for 24h, 4d, and 7d (Fig. 4a) or received an 24h Aβ-F pulse (Fig. 4b). At 24h, both cell types had engulfed and accumulated Aβ-F (Fig. 4c). Interestingly, in the co-culture set up, both cell types appeared to contain similar levels of Aβ deposits (Fig. 4d and e and Supplementary Figure 5). This result was different from the monocultures, where microglia contained much more Aβ than astrocytes (Fig. 3). At d7, the overall intracellular Aβ signal in the co-culture had significantly decreased, compared to the 24h time point (Fig. 4f). To investigate which cell type was responsible for the reduction, microglial and astrocytic Aβ were analyzed, respectively. At 7d, intracellular Aβ levels were reduced in both microglia (Fig. 4g) and astrocytes (Fig. 4h), compared to 24h. This reduction in Aβ deposits was not observed in the astrocytic monocultures (Fig. 3d). When we analyzed the cells that received an Aβ-F pulse, we noted that the microglia cells contained more Aβ inclusions at 24h+3d, compared to the astrocytes (Fig. 4i–k), indicating that the presence of microglia either increases the degradation capacity of the astrocytes or causes astrocytes to dispose of their inclusions via secretion or cell-to-cell transfer.

### Microglia degrade aggregated αSYN and Aβ better than astrocytes

**Fig. 5:**
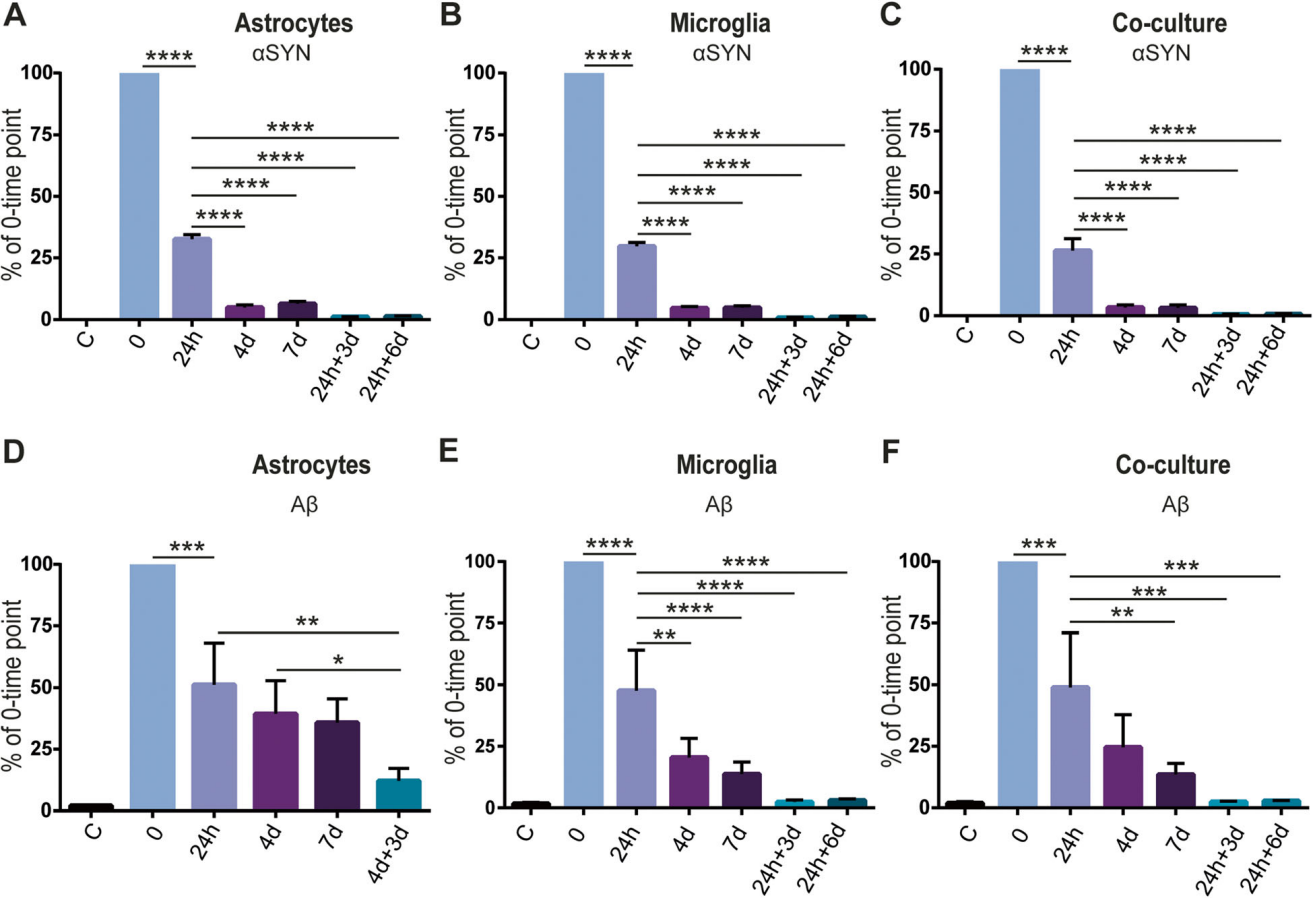
Astrocytes and microglia clear equal amounts of αSYN, but microglia clear extracellular Aβ more effectively. αSYN levels in the medium revealed that both astrocytes and microglia engulfed almost 75% of the initial αSYN. Furthermore, both astrocytes and microglia continued to ingest αSYN during the 7d of exposure. Secretion of αSYN was observed in all cultures at 24h+3d and 24h+6d (**a**–**c**). Analysis of Aβ in the medium revealed that astrocytes were less efficient in engulfing Aβ, compared to microglia. In the co-culture set-up, cells engulfed Aβ at the same level as in the microglial culture. Also, low secretion of Aβ could be observed at 24h+3d and 24h+6d in the microglia and co-cultures, whereas higher Aβ levels was observed at 4d+3d in the astrocyte cultures (**d**–**f**)

Both the glial monocultures and co-cultures are heterogeneous, containing cells of various size and shape. We noted that in small cells, the inclusions were often more densely packed, while large cells displayed scattered deposits (Figs. 1, 2, 3, and 4). However, we were unable to identify cells with no intracellular deposits. In order to study how much of the αSYN-F/Aβ-F that were internalized by the two cell types, we next analyzed the conditioned medium at different time points, using ELISA. After 24h exposure, almost 75% of the added αSYN had been cleared from the medium in all cell cultures, indicating that astrocytes and microglia are equally effective in ingesting αSYN-F (Fig. 5a–c). Furthermore, the αSYN levels were gradually reduced over 7d, suggesting that both astrocytes and microglia continuously take up new αSYN-F independent of their intracellular αSYN load. Together with the finding that intracellular αSYN increased over time in astrocytes, but remained unchanged in microglia cells, our results suggest that microglia degrade αSYN-F more effectively than astrocytes (at least when cultured separately). Interestingly, low but stable levels of αSYN could be detected in the medium at 24h+3d and 24h+6d, indicating that the cells secrete some of their intracellular αSYN into the medium (Fig. 5a–c).

Measurements of Aβ levels in the medium (Fig. 5d–f) showed that astrocytes and microglia engulfed similar amounts of the Aβ-F during the first 24h. However, while microglia cleared Aβ from the medium also after the 24h, astrocytes showed no significant uptake of Aβ-F between 24h to 7d, indicating that the intracellular Aβ load affects their phagocytic capacity. At 4d + 3d, higher Aβ levels were detected in the astrocyte medium compared to the microglia and the co-culture medium, suggesting that the astrocytes also secrete more Aβ than the microglia (Fig. 5d–f).

Next, we analyzed the levels of various cytokines in the cell culture medium using a cytokine array (Supplementary Figure 6). Our data indicate that the exposure to aggregated αSYN or Aβ had very little effect on the cytokine profile.

### Ingested αSYN enters the endo-lysosomal pathway and remains there

**Fig. 6:**
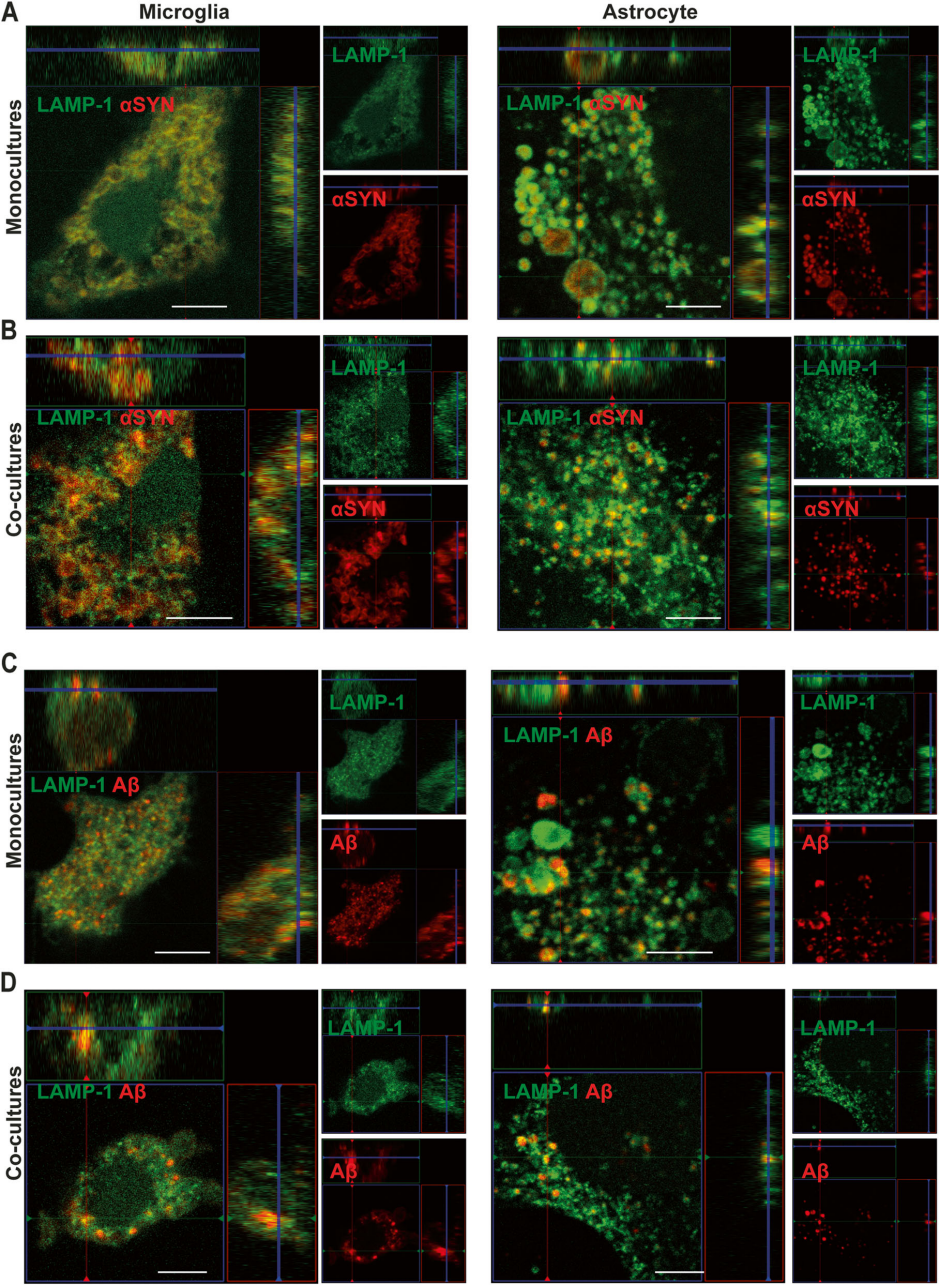
Ingested αSYN-F and Aβ-F are located in LAMP1+ vesicles. Confocal microscopy demonstrated that intracellular deposits of αSYN (**a**, **b**) and Aβ (**c**, **d**) were surrounded by LAMP1+ vesicles in both monocultures of astrocytes and microglia (**a** and **c**) and in the co-cultures (**b** and **d**). Scale bars = 20μm

In our previous studies of human astrocytes, we have shown that αSYN-F enters the endo-lysosomal pathway following ingestion [24]. To investigate if αSYN-F and Aβ-F also enters the endo-lysosomal pathway in microglia, the cells were stained for LAMP-1. By 24h, the majority of intracellular αSYN/Aβ were surrounded by LAMP-1 in both cell types (Fig. 6a–d). The pattern was identical in microglia and astrocytes cultured separately or in co-culture. Furthermore, the protein aggregates remained in the lysosomal compartments at all-time points, regardless of cell type and culture system (Supplementary Figure 7), suggesting that the αSYN-F/Aβ-F is cleared predominantly by lysosomal degradation and do not end up in other cellular compartments. In addition to LAMP-1, the microglial cultures were stained for CD68, which showed a very similar expression pattern. No change in the intensity of the CD68 staining was detected following αSYN or Aβ exposure (Supplementary Fig 8).

### Astrocytes transfer αSYN aggregates to microglia via secretory mechanisms

**Fig. 7:**
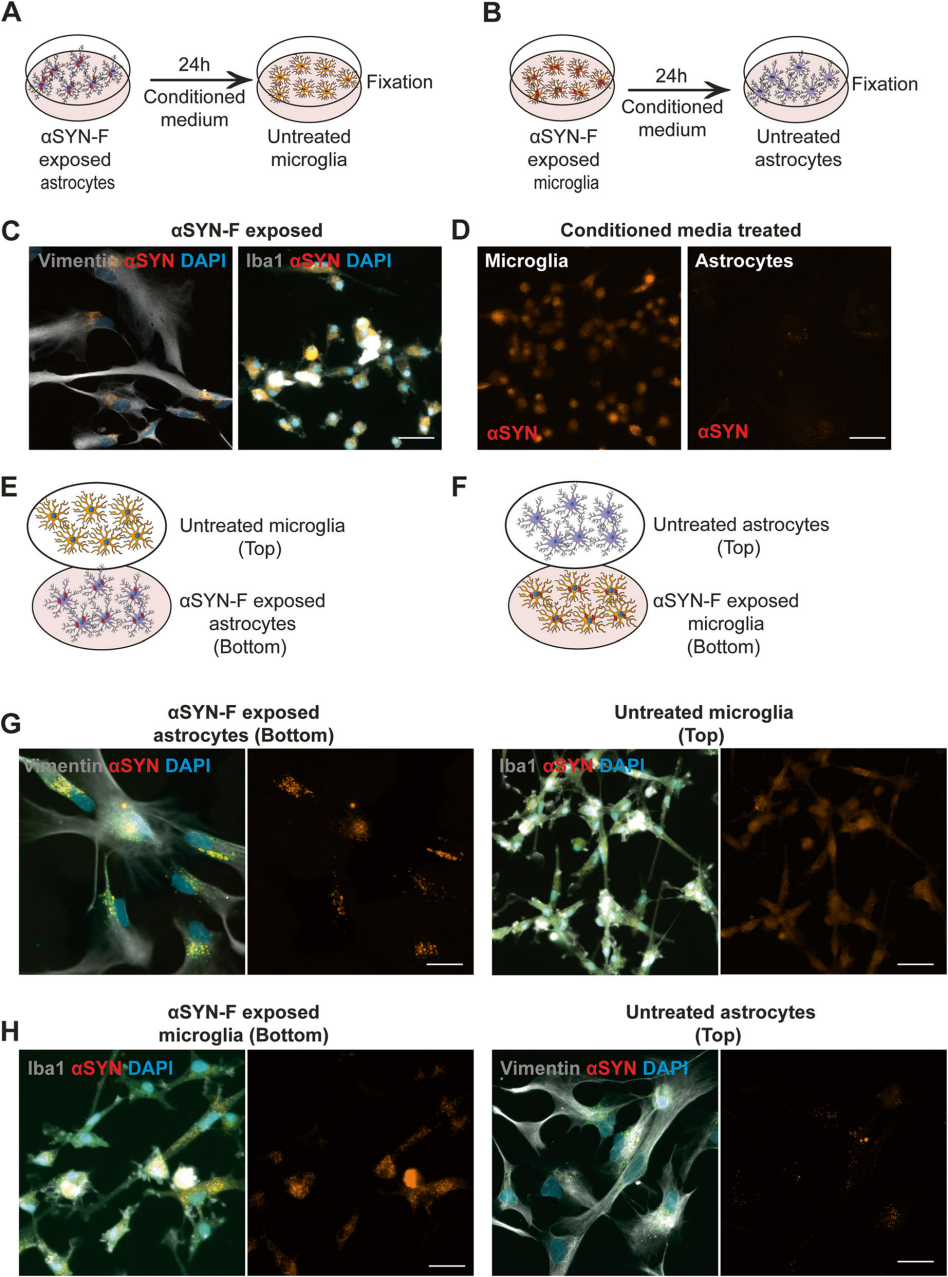
Secreted αSYN aggregates are transferred from astrocytes to microglia. Conditioned media experiments were performed to investigate if transfer of αSYN occurred from one cell type to another via secretion (**a**, **b**). Microglia that received conditioned medium from αSYN-exposed astrocytes contained high levels of αSYN, while astrocytes that received microglia medium had very little intracellular αSYN (**c**, **d**). To further study secretory spreading of αSYN aggregates between the two cell types, we performed experiments using a customized close-culture chamber, in which the astrocytes and microglia were cultured face-to-face, separated by a <1-mm space. In the chamber, either untreated microglia were co-cultured with αSYN-exposed astrocytes (**e**) or untreated astrocytes were co-cultured with αSYN-exposed microglia (**f**). Consistently with the conditioned media data, astrocytes were found to transfer a large proportion of their internalized αSYN to microglia (**g**), while microglia hardly transferred any αSYN to the astrocytes (**h**). Scale bars = 20μm

The observed reduction in astrocytic αSYN, when co-cultured with microglia, raised the possibility of active transfer of αSYN between the glia populations either via a secretion and uptake pathway or through direct cell-to-cell contact. Accordingly, we first performed conditioned media experiments (Fig. 7a, b). We observed that microglia internalized large amounts of αSYN, when treated with conditioned medium from αSYN-exposed astrocytes (Fig. 7c, d). In contrast, astrocytes treated with conditioned media from αSYN-exposed microglia had very low levels of intracellular αSYN (Fig. 7d). To further verify the diverse spreading of αSYN from astrocytes to microglia and vice versa, we performed experiments with a customized “close-culture” cell chamber (Supplementary Figure 9), in which the apical surfaces of astrocyte and microglia monocultures were separated by a <1 mm space. This space served as a common medium reservoir, which permitted the paracrine exchange of material, but prevented direct contact between the different cell-types (Fig. 7e, f). Our results confirm that astrocytes transfer a large proportion of their intracellular αSYN to microglia via secretory mechanisms, while microglia hardly transfer any αSYN to the astrocytes (Fig. 7g, h).

### Extensive contact between astrocytes and microglia enable cell-to-cell transfer of aggregated αSYN

**Fig. 8:**
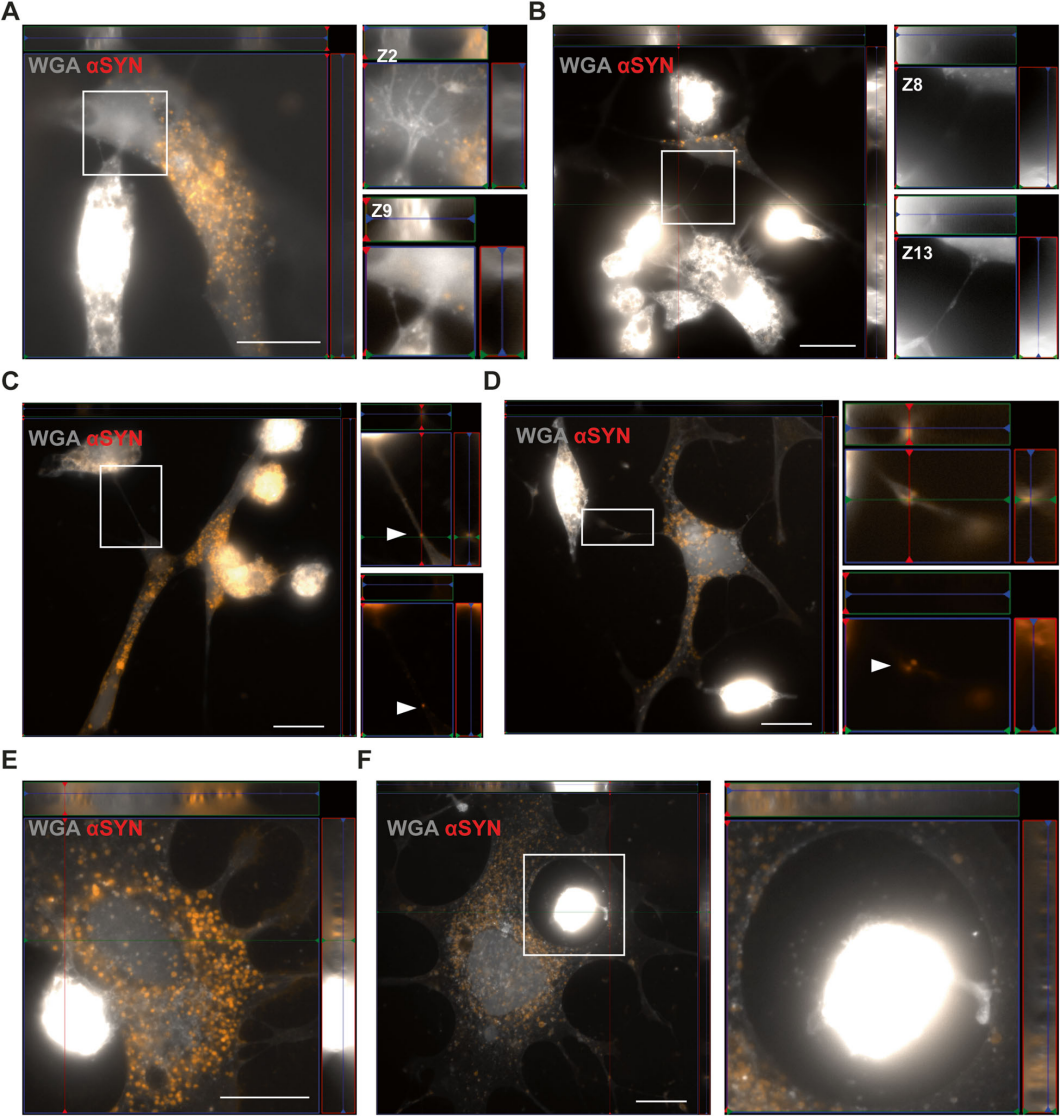
Direct contact between microglia and astrocytes enables cell-to-cell transfer of αSYN deposits. Astrocytes and microglia were found to have direct TNTs (**a**, **b**). Z-stacks of the zoomed regions of **a** and **b** are shown beside respective image. High resolution imaging revealed TNT-mediated transfer of αSYN between astrocytes and microglia (**c**, **d**, white arrows). The microglia were also found to be in close contact with (**e**) or completely surrounded by astrocytic membranes (**f**). Scale bars= 20μm

**Fig. 9:**
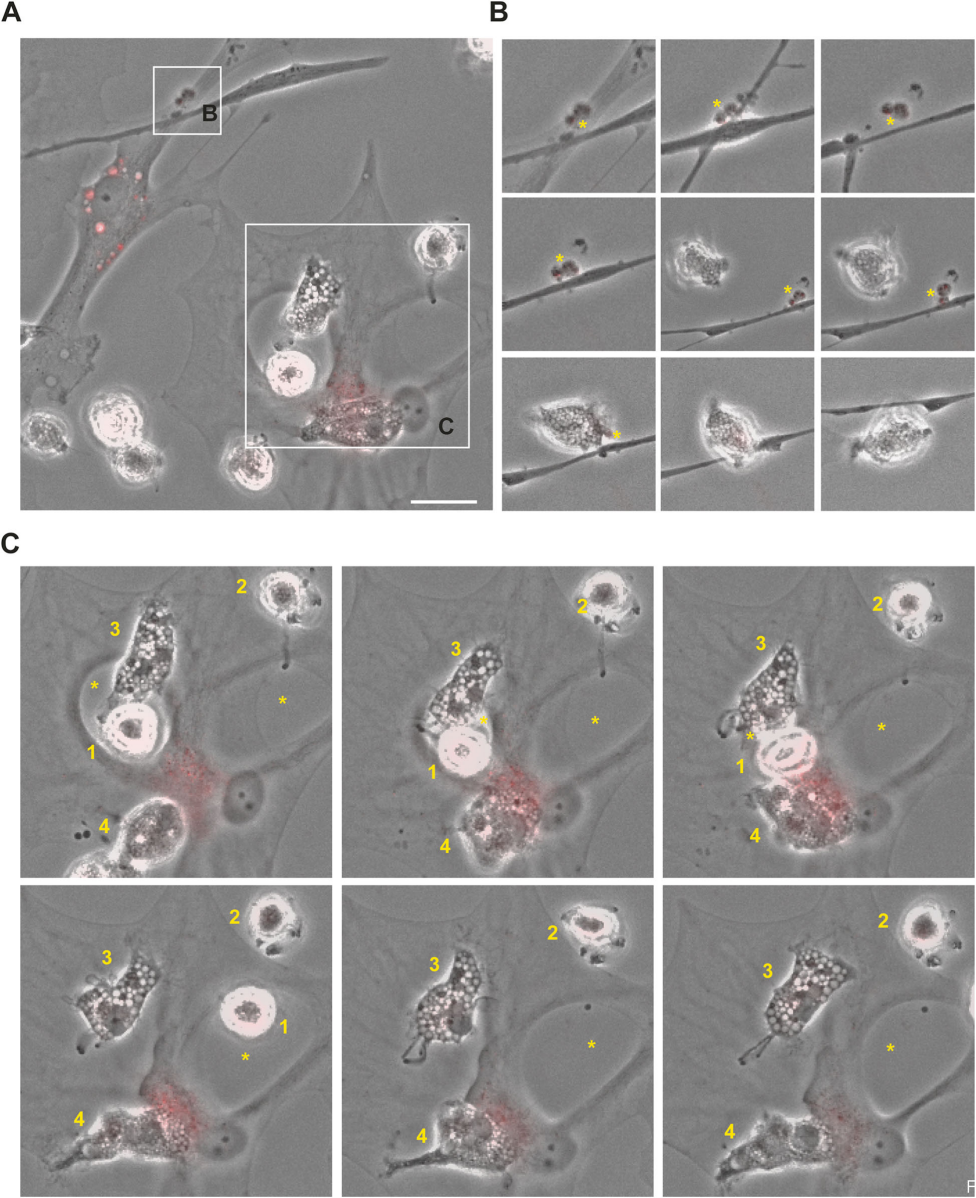
Microglia can attract and clear intracellular αSYN deposits from astrocytes via membrane contact. Time-lapse microscopy illustrated a complex interplay between astrocytes and microglia (**a**). Different time points of the regions of interest are shown in **b** and **c**. For **b,** the total duration from the first to last image is 10h and for **c** 11h. Astrocytes secreted large αSYN-containing vesicles (yellow star) that were ingested by microglia (**b**). Moreover, microglia were found to be in direct contact with astrocytes in ring-shaped areas (yellow star) encapsulated by astrocytic membrane (microglia 1) or attached to the cell membrane of this area (microglia 2) (**c**). Microglia were anchored to the astrocyte membrane via distinct protrusions (microglia 2 and 3) and often interacted with the astrocytes in the region where the most intracellular αSYN deposits were situated (microglia 4). During this interaction, the deposits appeared to decrease dramatically, indicating direct αSYN transmission followed by instant degradation. Scale bar= 10μm

Subsequently, we analyzed whether the astrocytes and microglia are in direct contact, as this could be another potential mechanism to spread αSYN from one cell to another. Indeed, multiple TNTs were formed between the astrocytes and microglia in the co-culture (Fig. 8a, b). Notably, deposits of aggregated αSYN could be found within the TNTs, indicating TNT-mediated transfer (Fig. 8c, d). We also observed frequent membrane contact between astrocytes and microglia (Fig. 8e). Often, the microglial cells were found within a ring-shaped area, completely surrounded by an astrocytic membrane. These microglia contacted the surrounding astrocyte with sprouting extensions (Fig. 8f). Live cell imaging shed further light on the complex crosstalk between astrocytes and microglia (Fig. 9a). We noticed that large αSYN-containing vesicles that were secreted from the astrocytes were effectively removed by the patrolling microglia (Fig. 9b). As previously, we found microglial cells encircled by astrocytic membranes (Fig. 9c), and in contact with the surrounding astrocyte via sprouting extensions (Fig. 9c). Interestingly, whenever this cellular arrangement occurred, the αSYN deposits in the astrocytes, which was prominently located around the nucleus, was shifted to the side of the interacting microglia (Fig. 9c). We also noted that the αSYN load in the astrocyte was dramatically reduced during the microglia interaction, indicating that microglia attract and clear intracellular protein deposits directly from the astrocytes (Fig. 9c).

## Discussion

Growing evidence indicates that insufficient degradation of protein aggregates and chronic inflammation are driving forces of AD and PD brain pathology [30–32]. Consequently, the role of glial cells in disease progression has received much attention lately. Microglia and astrocytes are known to be responsible for many inflammatory processes in affected brains, including phagocytosis of αSYN and Aβ aggregates, secretion of inflammatory mediators, and stimulation of the innate immune system [8–12]. In order to develop effective therapies, it is of great importance to understand how the two glial cell types communicate and coordinate their responses to αSYN and Aβ pathology. We have previously shown that astrocytes accumulate large amounts of αSYN and Aβ aggregates, resulting in severe cellular stress [11, 12, 23, 24]. The aim of the present study was to compare uptake and accumulation of αSYN and Aβ in human astrocytes and microglia, when cultured separately or in a co-culture set up. Moreover, we sought to identify specific mechanisms by which the two cell types crosstalk in regard to αSYN and Aβ pathology.

Our data show that in the separate cultures, both microglia and astrocytes ingest αSYN and Aβ aggregates, but microglia appear to be more effective in degrading the protein aggregates. In a recent publication, we showed that astrocytes, but not microglia, have the capacity to act as antigen-presenting cells (APCs) when exposed to αSYN fibrils [24]. Accordingly, astrocytes could be found in very close proximity to infiltrating T cells in the PD brain [24]. One of the features of APCs is the ability to degrade antigens slowly in order to present them on the surface for T cells. These observations gave rise to the idea that microglia may be primarily responsible for degrading protein aggregates, whereas astrocytes may act as a bridge between the brain and the peripheral immune system. This idea has further support from the fact that astrocytes are essential for the formation and maintenance of the blood-brain barrier.

In our co-culture experiments, we could observe that, even though both cell types engulfed αSYN and Aβ fibrils, microglia were responsible for a greater uptake of the aggregates. Interestingly, astrocytes accumulated less αSYN and Aβ deposits in the co-cultures, compared to the monocultures. A recent study showed that astrocytes and microglia degrade dead cells in a very organized and specialized manner; microglia degrade neuronal dendrites, cell bodies, and nuclei while astrocytes phagocytose apoptotic bodies that are released from the dying neurons [10]. These data suggest that microglia and astrocytes could share the burden by dividing toxic materials in a size-dependent manner, where microglia would phagocytose bigger aggregates and astrocytes smaller aggregates. These observations are in line with our previous discoveries that astrocytes easily degrade αSYN and Aβ monomers, while they accumulate larger oligomers and fibrils [11, 12]. Hence, it is possible that astrocytes engulf less protein aggregates in the presence of microglia and thereby allow better degradation of the ingested material. Damisah et al. provide support for this in their recent study, where microglia were eliminated from mice using a CSF1R antagonists, after which neuronal cell death was induced. They found that astrocytes engulfed the material which was intended for microglia but degraded it at a slower rate [10]. An accumulating body of evidence emphasizes astrocytes as one of the major secretory cells in the CNS, responsible for secretion of neurotransmitters, growth factors, inflammatory mediators, and toxic proteins [33]. We have previously shown that an incomplete degradation of Aβ_42_ protofibrils by astrocytes results in the release of extracellular vesicles containing N-truncated, neurotoxic Aβ [12]. Here, we observed that microglia phagocytosed large amounts of αSYN from astrocytic conditioned medium, whereas very little αSYN could be detected in astrocytes that had been treated with microglial-conditioned medium. Based on these results and previous studies, we hypothesize that astrocytes secrete some of the ingested αSYN and Aβ, which is then phagocytosed by the surrounding microglia. Several studies have demonstrated that astrocytes are capable of stimulating microglia phagocytosis by secretion of cytokines such as IL33 and the complement protein C3 [19, 34]. Therefore, investigating what inflammatory molecules astrocytes and microglia secrete when co-cultured would provide valuable insights into how they crosstalk.

Our previous results indicate that astrocytes are capable of transferring αSYN as well as MHCII to other astrocytes via direct membrane contact and through TNTs, implicating astrocytes as one of the important players in propagation of αSYN pathology [11, 24]. In the co-culture experiments in this study, we found that astrocytes also establish direct contact with microglia and that the two glial cell types remained in a continual alliance for a long time, as shown by immunocytochemistry and time-lapse microscopy. Notably, we could for the first time show that astrocytes and microglia are frequently connected via TNTs. The presence of αSYN deposits within the TNTs suggest that these structures are used for cell-to-cell transfer of protein aggregates. Moreover, microglia were often situated in ring-shaped areas, “in the middle” of astrocytes, clearly anchored to the astrocyte membrane via extending protrusions. As shown with our time-lapse recordings, the αSYN-deposits appeared to be immediately degraded, after being transferred from astrocytes to microglia. These findings are intriguing, but further studies using for example multi-photon microscopy are needed to confirm the phenomenon in the living brain.

## Conclusions

In conclusion, our results demonstrate that there is a synergistic effect of astrocytes and microglia in processing of Aβ and αSYN aggregates, with relevance to AD and PD pathogenesis. Mainly, this interplay between the two cell types involves transfer of protein aggregates from astrocytes to microglia, which may be an important mechanism for the clearance of protein aggregates within affected brains. However, it may also constitute a risk for spreading the pathology within and between interconnected networks. Taken together, our results highlight the importance of microglia-astrocyte crosstalk with respect to AD and PD pathology. Apart from increasing our knowledge on the underlying disease mechanisms, these insights may be of great relevance for identifying new therapeutic targets for these neurodegenerative disorders.

## Supplementary Information

**Additional file 1:.** Supplementary Figure 1. Astrocytes proliferate during the time course of the experiment. Quantification of the number of viable cells per field demonstrated that the astrocytes proliferate during the 7d period.

**Additional file 2:.** Supplementary Figure 2. Microglia proliferate during the time course of the experiment. Quantification of the number of viable cells per field demonstrated that the microglia proliferate during the 7d period.

**Additional file 3:.** Supplementary Figure 3. Characterization of astrocyte and microglia cultures. Human iPSC derived astrocytes expressed the astrocytic markers GFAP, ALDH1L1, vimentin and S100β (A). Human iPSC derived microglia expressed Iba1 and P2Y12 (B).

**Additional file 4:.** Supplementary Figure 4. αSYN accumulation is reduced when microglia and astrocytes are co-cultured. The separate channels from Figure 2E. Scale bar = 20 μm.

**Additional file 5:.** Supplementary Figure 5. Intracellular Aβ is reduced when astrocytes and microglia are cultured together. The separate channels from Figure 4E. Scale bar = 20 μm.

**Additional file 6:.** Supplementary Figure 6. Unchanged cytokine profile following αSYN or Aβ exposure. Cytokine array data indicate that exposure to aggregated αSYN or Aβ had very little effect on the cytokine profile of astrocytes and microglia, both in monocultures and in co-cultures. Measurements of three independent experiments did not reveal any significant differences between the cultures.

**Additional file 7:.** Supplementary Figure 7. Ingested αSYN is located in LAMP1+ vesicles. Intracellular deposits of αSYN was surrounded by LAMP1+ vesicles at all-time points in both separate cultures of astrocytes (A) and microglia (B) and in co-cultures (C). Close-up images of the white rectangles are shown below. Scale bars = 20μm.

**Additional file 8:.** Supplementary Figure 8. Expression of CD68 in microglia following αSYN or Aβ exposure. The myeloid-specific endo-lysosomal marker, CD68, was expressed by the microglia. No change in the intensity of the CD68 staining was detected over time or following αSYN or Aβ exposure. Scale bars = 20μm.

**Additional file 9:.** Supplementary Figure 9. Close-culture chamber device. Exploded view of the close-culture chamber system consisting of interlocking top and bottom steel rings, and two coverslips (A). Top view of the assembled close-culture chamber, indicating the position of 3 access ports, which facilitate medium exchange, and the outer (32 mm) and inner (15 mm) diameters of the rings (B). Cross-sectional cutaway view of the assembled close-culture chamber. The enlarged area illustrates the width (1.75 mm) of the flange on which the upper coverslip rests, and the 1 mm open space between the sandwiched coverslips (C). Monocultures are established on each of the coverslips, and when assembled in the device the apical surfaces of cell-type 1 (e.g. astrocytes) and cell-type 2 (e.g. microglia) are separated by a gap of < 1 mm, but share a common reservoir of growth medium (D).

## Data Availability

All data used and analyzed for the current study are available from the corresponding author on reasonable request.

## Abbreviations

αSYN: Alpha-synuclein
αSYN-F: Alpha-synuclein fibrils
AD: Alzheimer’s disease
Aβ: Amyloid-beta
Aβ-F: Amyloid-beta fibrils
APC: Antigen-presenting cells
bFGF: Basic fibroblast growth factor
CNTF: Ciliary neurotrophic factor
IGF-1: Insulin-like growth factor 1
HPC: Hematopoietic stem cell
iPSCs: Induced pluripotent stem cells
NES: Neuroepithelial-like stem cells
PD: Parkinson’s disease
TNT: Tunneling nanotubes
