# Supplementary figures and images for: Crosstalk between astrocytes and microglia results in increased degradation of α-synuclein and amyloid-β aggregates
Source: J Neuroinflammation. 2021 Jun 3;18:124. doi: 10.1186/s12974-021-02158-3 (PMC8173980; doi:10.1186/s12974-021-02158-3)

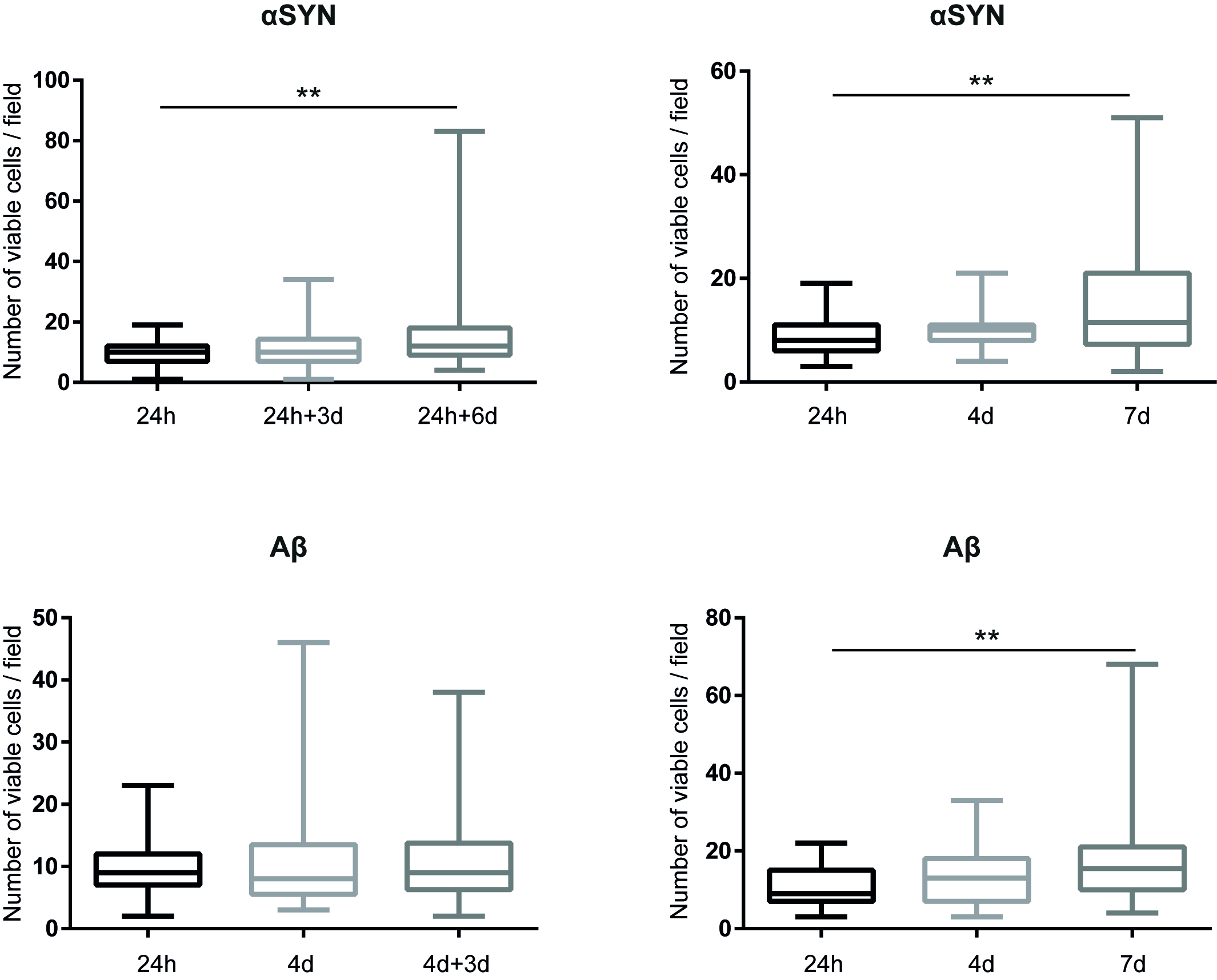

Supplement: Supplementary file 1 — Additional file 1:. Supplementary Figure 1. Astrocytes proliferate during the time course of the experiment. Quantification of the number of viable cells per field demonstrated that the astrocytes proliferate during the 7d period. [file 12974_2021_2158_MOESM1_ESM.tif]

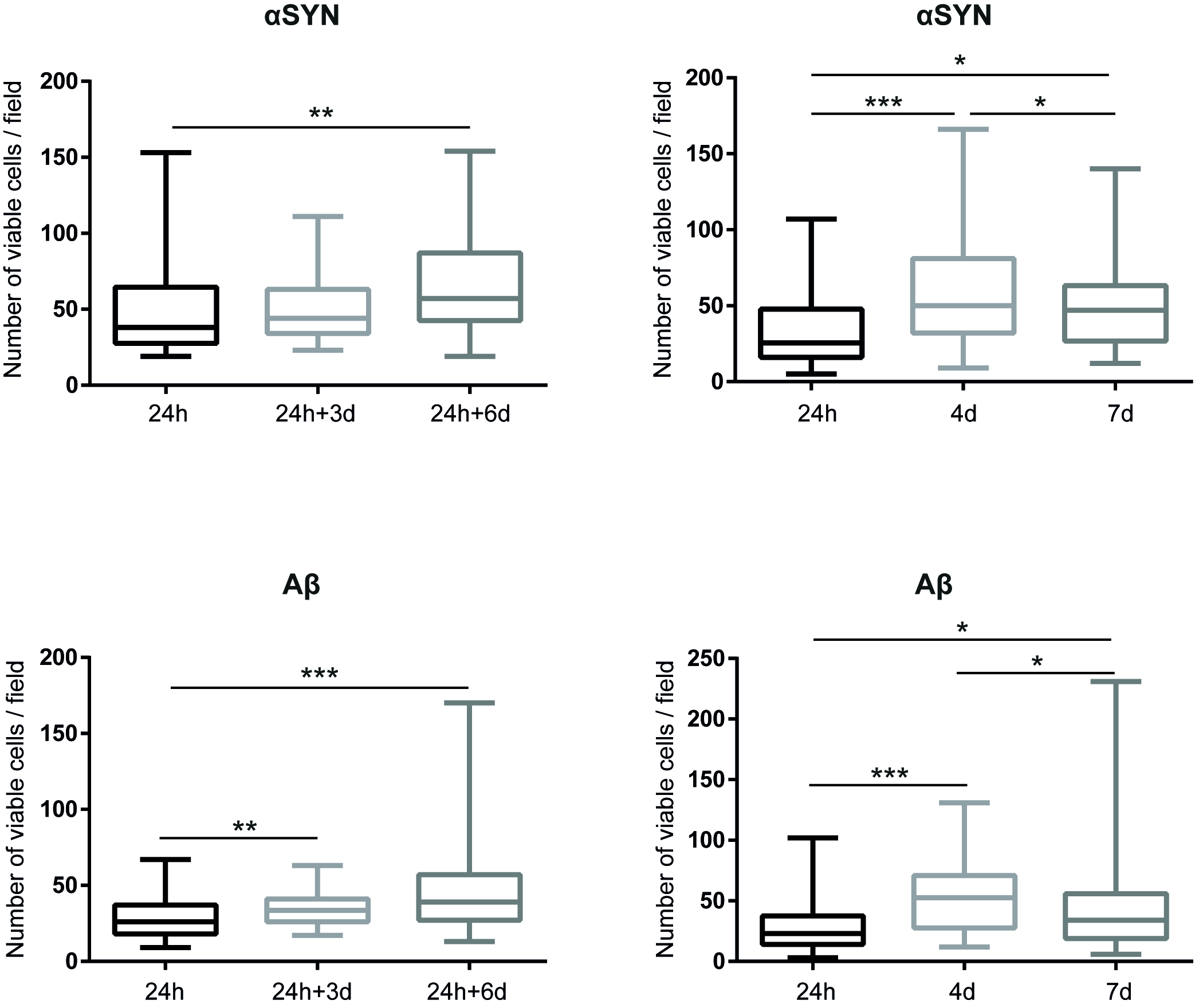

Supplement: Supplementary file 2 — Additional file 2:. Supplementary Figure 2. Microglia proliferate during the time course of the experiment. Quantification of the number of viable cells per field demonstrated that the microglia proliferate during the 7d period. [file 12974_2021_2158_MOESM2_ESM.tif]

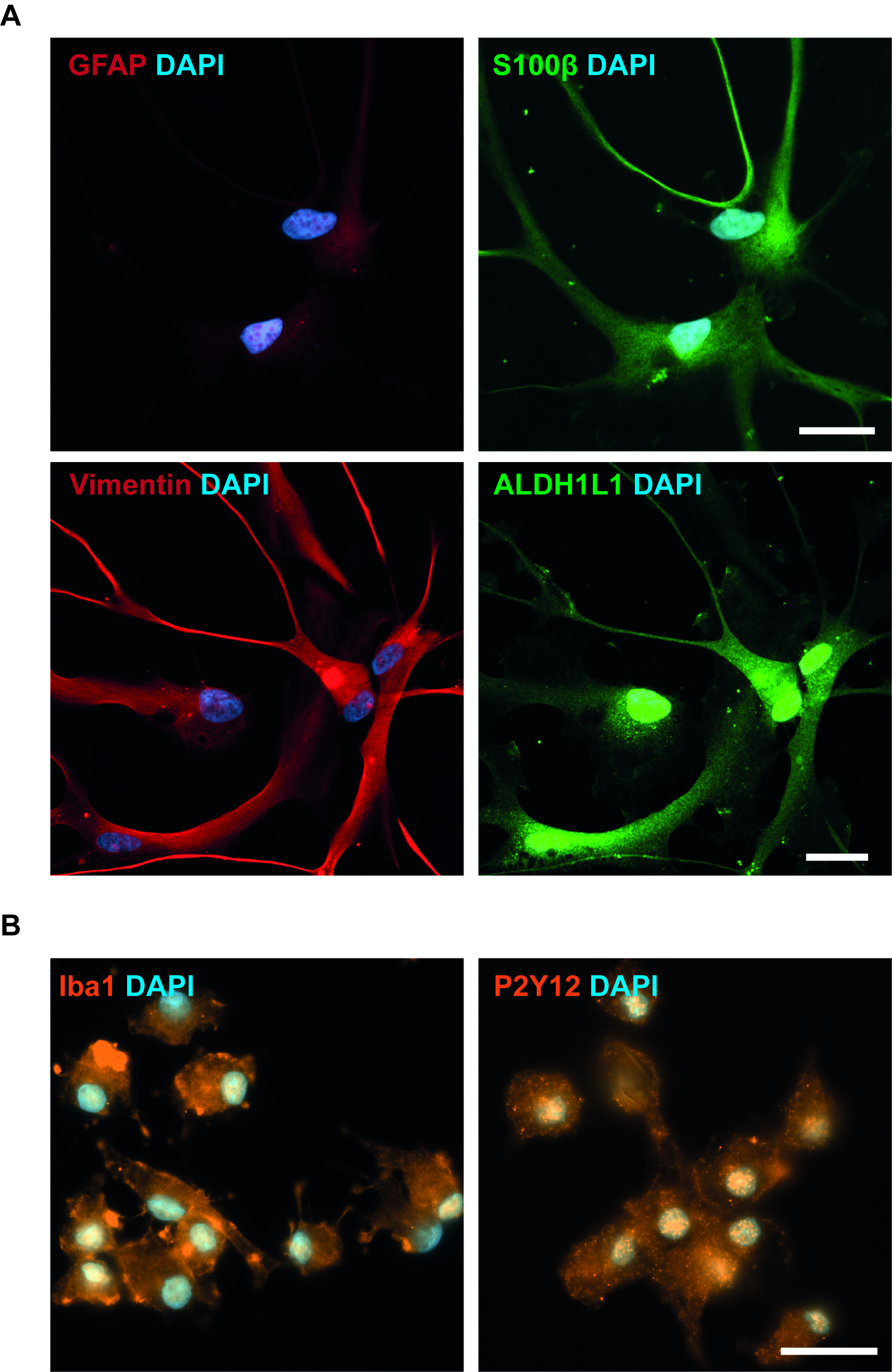

Supplement: Supplementary file 3 — Additional file 3:. Supplementary Figure 3. Characterization of astrocyte and microglia cultures. Human iPSC derived astrocytes expressed the astrocytic markers GFAP, ALDH1L1, vimentin and S100β (A). Human iPSC derived microglia expressed Iba1 and P2Y12 (B). [file 12974_2021_2158_MOESM3_ESM.tif]

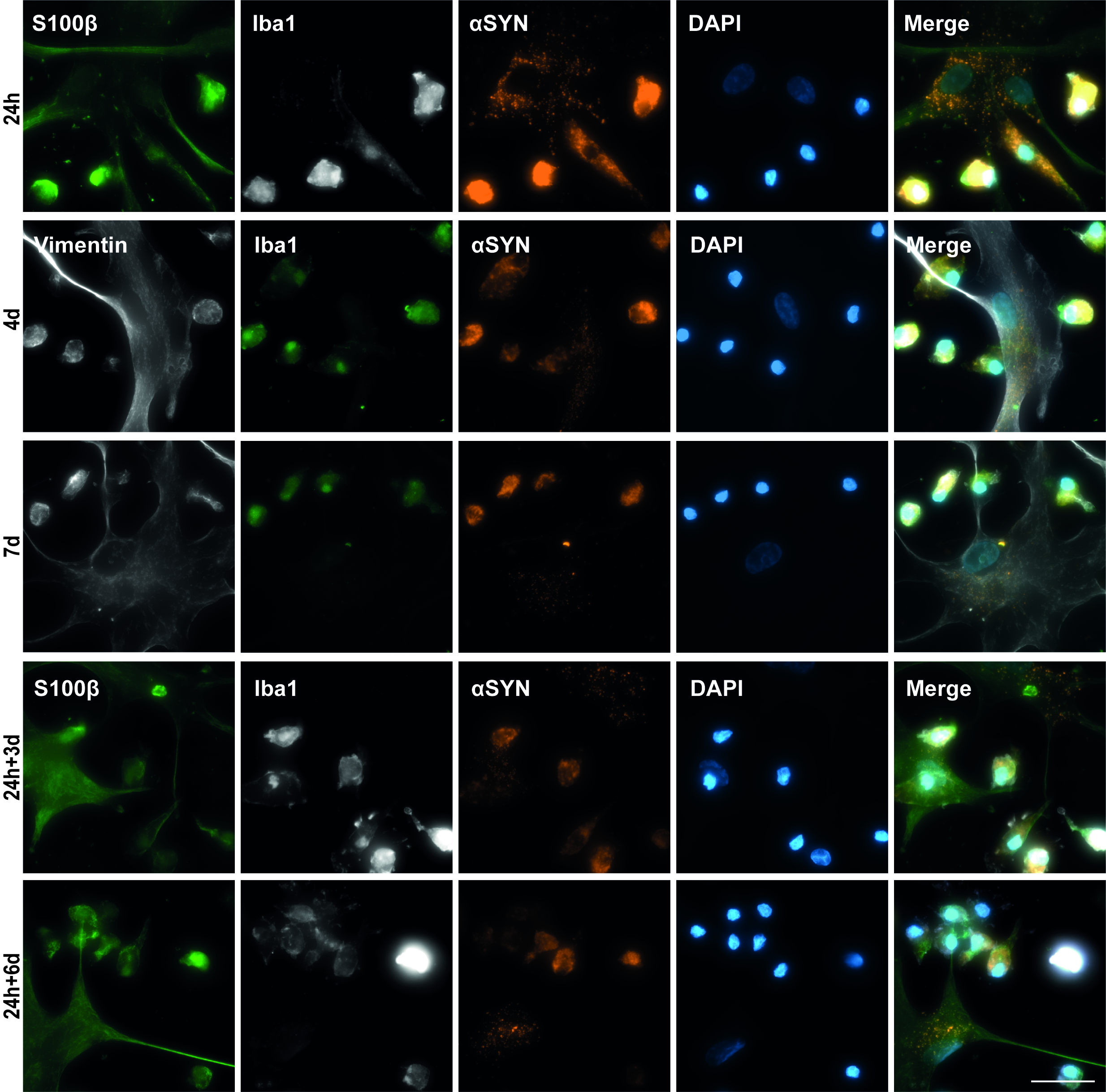

Supplement: Supplementary file 4 — Additional file 4:. Supplementary Figure 4. αSYN accumulation is reduced when microglia and astrocytes are co-cultured. The separate channels from Figure 2E. Scale bar = 20 μm. [file 12974_2021_2158_MOESM4_ESM.tif]

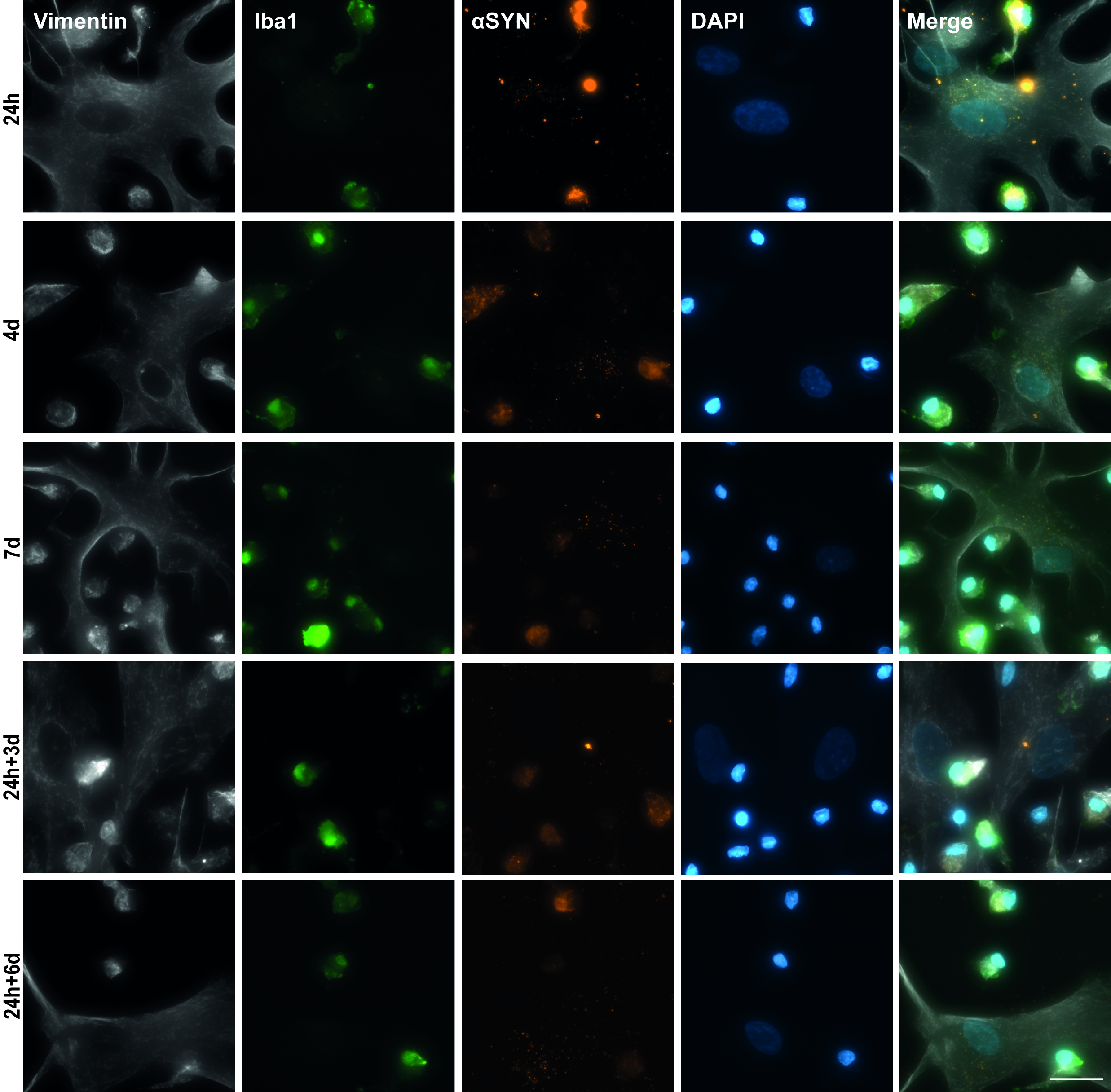

Supplement: Supplementary file 5 — Additional file 5:. Supplementary Figure 5. Intracellular Aβ is reduced when astrocytes and microglia are cultured together. The separate channels from Figure 4E. Scale bar = 20 μm. [file 12974_2021_2158_MOESM5_ESM.tif]

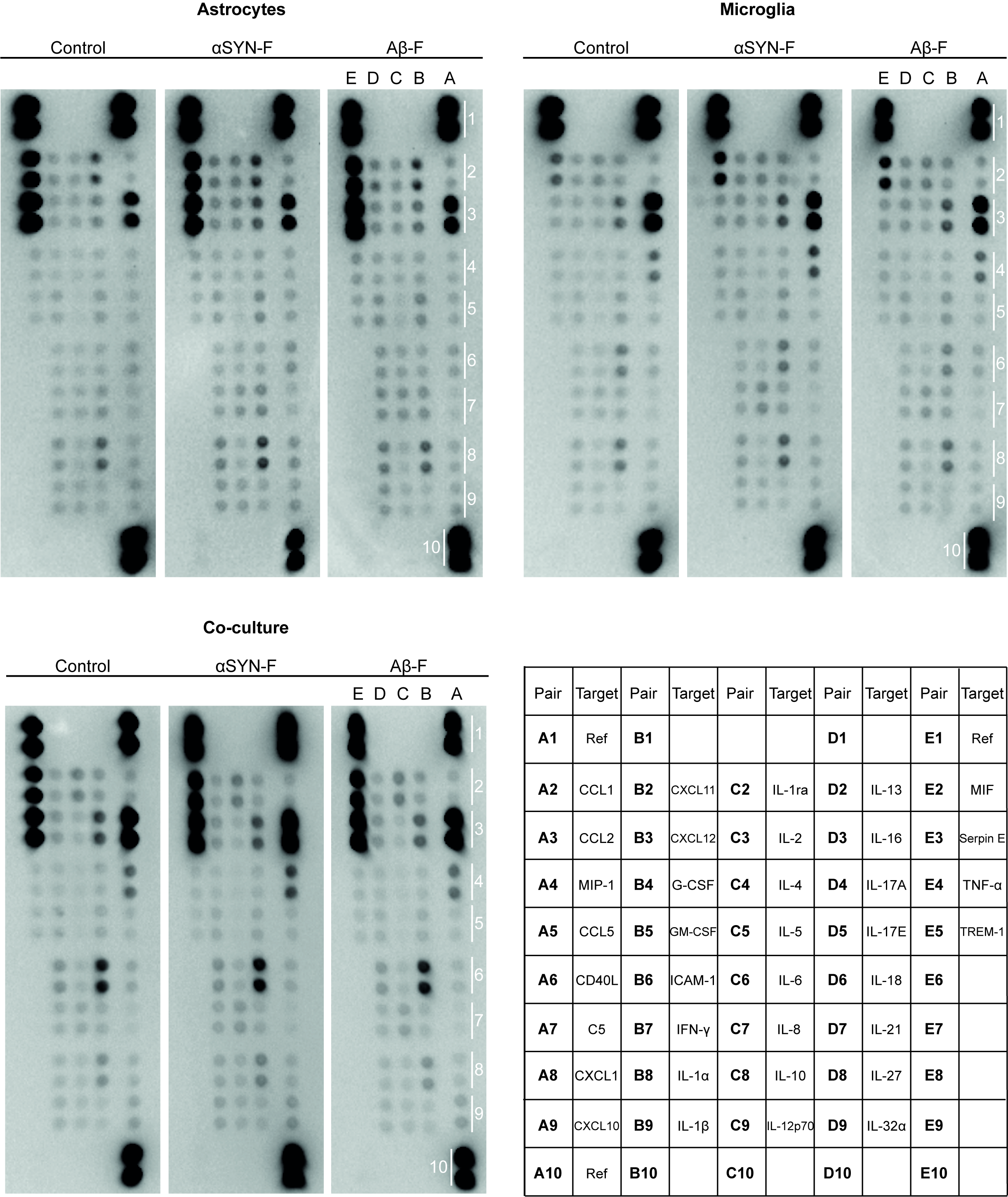

Supplement: Supplementary file 6 — Additional file 6:. Supplementary Figure 6. Unchanged cytokine profile following αSYN or Aβ exposure. Cytokine array data indicate that exposure to aggregated αSYN or Aβ had very little effect on the cytokine profile of astrocytes and microglia, both in monocultures and in co-cultures. Measurements of three independent experiments did not reveal any significant differences between the cultures. [file 12974_2021_2158_MOESM6_ESM.tif]

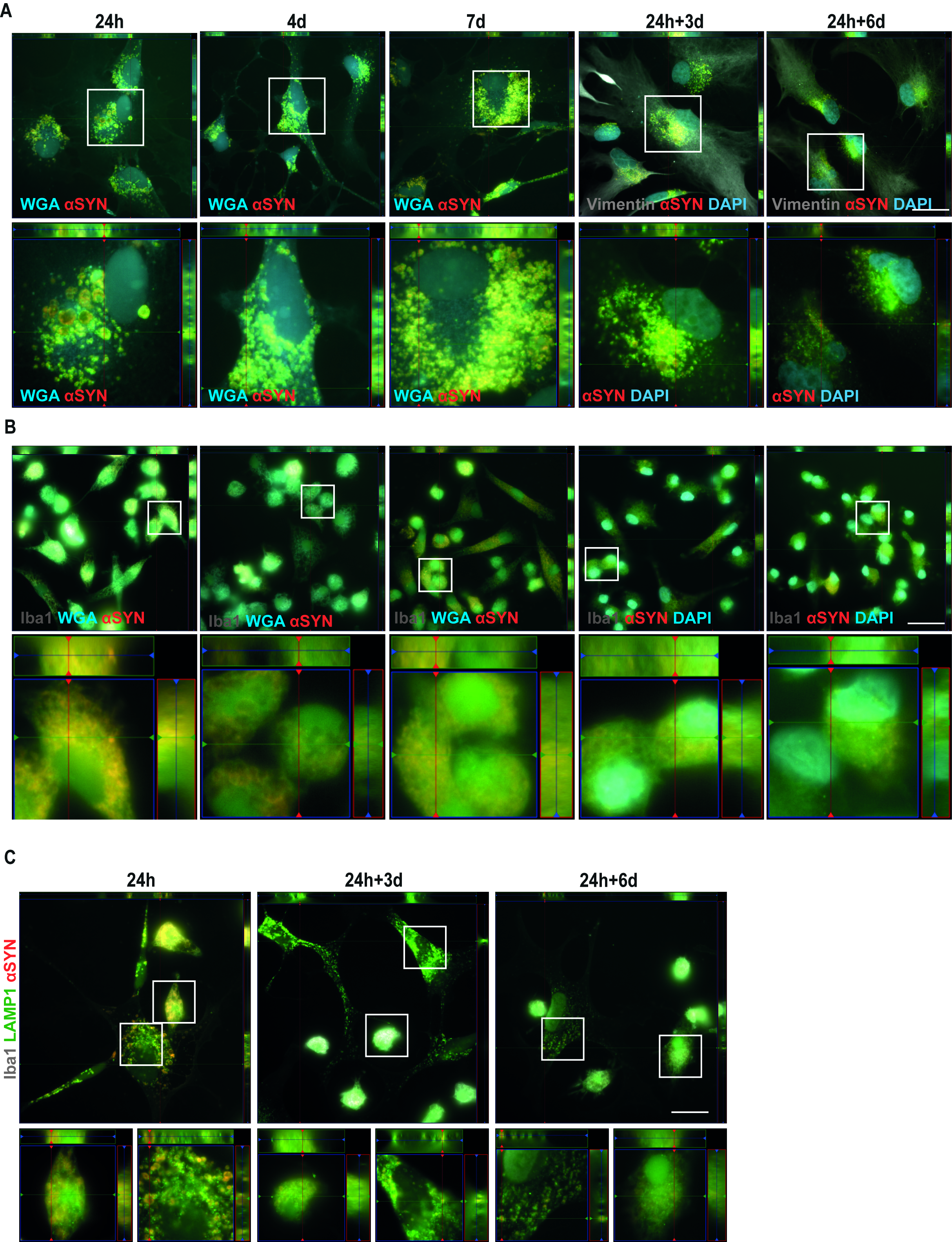

Supplement: Supplementary file 7 — Additional file 7:. Supplementary Figure 7. Ingested αSYN is located in LAMP1+ vesicles. Intracellular deposits of αSYN was surrounded by LAMP1+ vesicles at all-time points in both separate cultures of astrocytes (A) and microglia (B) and in co-cultures (C). Close-up images of the white rectangles are shown below. Scale bars = 20μm. [file 12974_2021_2158_MOESM7_ESM.tif]

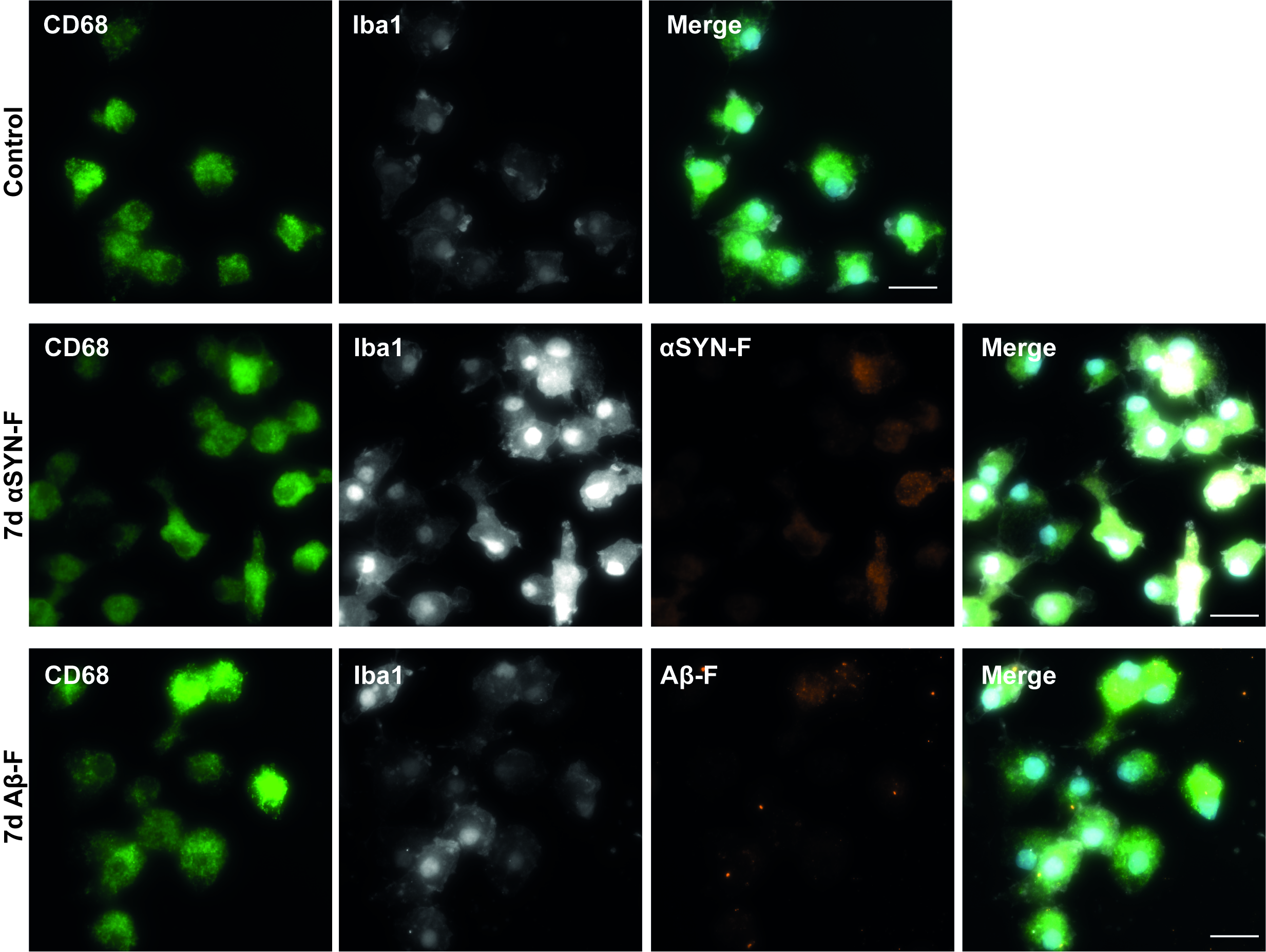

Supplement: Supplementary file 8 — Additional file 8:. Supplementary Figure 8. Expression of CD68 in microglia following αSYN or Aβ exposure. The myeloid-specific endo-lysosomal marker, CD68, was expressed by the microglia. No change in the intensity of the CD68 staining was detected over time or following αSYN or Aβ exposure. Scale bars = 20μm. [file 12974_2021_2158_MOESM8_ESM.tif]

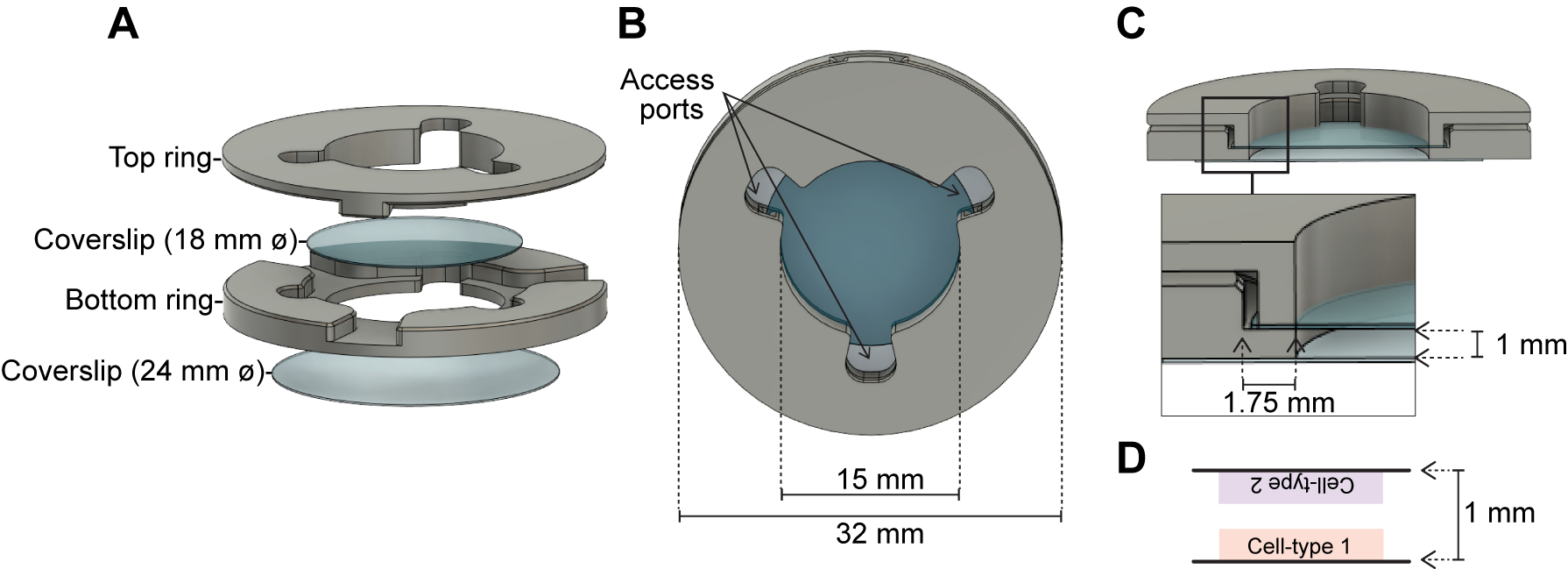

Supplement: Supplementary file 9 — Additional file 9:. Supplementary Figure 9. Close-culture chamber device. Exploded view of the close-culture chamber system consisting of interlocking top and bottom steel rings, and two coverslips (A). Top view of the assembled close-culture chamber, indicating the position of 3 access ports, which facilitate medium exchange, and the outer (32 mm) and inner (15 mm) diameters of the rings (B). Cross-sectional cutaway view of the assembled close-culture chamber. The enlarged area illustrates the width (1.75 mm) of the flange on which the upper coverslip rests, and the 1 mm open space between the sandwiched coverslips (C). Monocultures are established on each of the coverslips, and when assembled in the device the apical surfaces of cell-type 1 (e.g. astrocytes) and cell-type 2 (e.g. microglia) are separated by a gap of < 1 mm, but share a common reservoir of growth medium (D). [file 12974_2021_2158_MOESM9_ESM.tif]
